# Genomic Characterization of Skin and Soft Tissue Streptococcus pyogenes Isolates from a Low-Income and a High-Income Setting

**DOI:** 10.1128/msphere.00469-22

**Published:** 2022-12-12

**Authors:** Saikou Y. Bah, Alexander J. Keeley, Edwin P. Armitage, Henna Khalid, Roy R. Chaudhuri, Elina Senghore, Jarra Manneh, Lisa Tilley, Michael Marks, Saffiatou Darboe, Abdul K. Sesay, Thushan I. de Silva, Claire E. Turner

**Affiliations:** a School of Biosciences, The Florey Institute, University of Sheffield, Sheffield, United Kingdom; b Department of Infection, Immunity & Cardiovascular Disease, The Florey Institute, University of Sheffield Medical School, Sheffield, United Kingdom; c The Medical Research Council, The Gambia, London School of Hygiene and Tropical Medicine, Serekunda, The Gambia; d Department of Microbiology, Sheffield Teaching Hospitals NHS Foundation Trust, Sheffield, United Kingdom; e Clinical Research Department, Faculty of Infectious and Tropical Diseases, London School of Hygiene & Tropical Medicine, London, United Kingdom; f Hospital for Tropical Diseases, London, United Kingdom; The University of Texas Medical Branch at Galveston

**Keywords:** FCT region, bacterial genomics, *emm* types, group A *Streptococcus*, skin infection, vaccine

## Abstract

Streptococcus pyogenes is a leading cause of human morbidity and mortality, especially in resource-limited settings. The development of a vaccine against S. pyogenes is a global health priority to reduce the burden of postinfection rheumatic heart disease. To support this, molecular characterization of circulating S. pyogenes isolates is needed. We performed whole-genome analyses of S. pyogenes isolates from skin and soft tissue infections in Sukuta, The Gambia, a low-income country (LIC) in West Africa where there is a high burden of such infections. To act as a comparator to these LIC isolates, skin infection isolates from Sheffield, United Kingdom (a high-income country [HIC]), were also sequenced. The LIC isolates from The Gambia were genetically more diverse (46 *emm* types in 107 isolates) than the HIC isolates from Sheffield (23 *emm* types in 142 isolates), with only 7 overlapping *emm* types. Other molecular markers were shared, including a high prevalence of the skin infection-associated *emm* pattern D and the variable fibronectin-collagen-T antigen (FCT) types FCT-3 and FCT-4. Fewer of the Gambian LIC isolates carried prophage-associated superantigens (64%) and DNases (26%) than did the Sheffield HIC isolates (99% and 95%, respectively). We also identified streptococcin genes unique to 36% of the Gambian LIC isolates and a higher prevalence (48%) of glucuronic acid utilization pathway genes in the Gambian LIC isolates than in the Sheffield HIC isolates (26%). Comparison to a wider collection of HIC and LIC isolate genomes supported our findings of differing *emm* diversity and prevalence of bacterial factors. Our study provides insight into the genetics of LIC isolates and how they compare to HIC isolates.

**IMPORTANCE** The global burden of rheumatic heart disease (RHD) has triggered a World Health Organization response to drive forward development of a vaccine against the causative human pathogen Streptococcus pyogenes. This burden stems primarily from low- and middle-income settings where there are high levels of S. pyogenes skin and soft tissue infections, which can lead to RHD. Our study provides much needed whole-genome-based molecular characterization of isolates causing skin infections in Sukuta, The Gambia, a low-income country (LIC) in West Africa where infection and RHD rates are high. Although we identified a greater level of diversity in these LIC isolates than in isolates from Sheffield, United Kingdom (a high-income country), there were some shared features. There were also some features that differed by geographical region, warranting further investigation into their contribution to infection. Our study has also contributed data essential for the development of a vaccine that would target geographically relevant strains.

## INTRODUCTION

Streptococcus pyogenes (group A Streptococcus [GAS]) is a human-specific pathogen and a leading cause of morbidity and mortality, especially in resource-limited countries. S. pyogenes can cause diseases ranging from mild superficial infections, such as impetigo and pharyngitis, to invasive diseases, such as necrotizing fasciitis and streptococcal toxic shock syndrome (1), and can also cause postinfection autoimmune sequelae such as acute rheumatic fever (ARF) leading to rheumatic heart disease (RHD). There is a substantial global burden of RHD, accounting for approximately 320,000 deaths in 2015, the majority of which were recorded in sub-Saharan Africa (2). Recognizing this burden, the World Health Organization (WHO) has prioritized the need for a vaccine that would have global coverage and recommended an increase in research, especially in low- and middle-income countries (LMICs) (3).

Progress toward a vaccine for S. pyogenes has been hampered over the years by the association of the most promising vaccine candidate, the surface protein M, with the development of RHD. This may be circumvented by targeting the N-terminal portion of the M protein, but this region is hypervariable, and thus any vaccine would be serotype/genotype specific. S. pyogenes isolates are genotyped by sequencing the corresponding hypervariable 5′ region of the M protein-encoding gene, *emm*. Over 220 different *emm* types have been identified globally, but in high-income countries (HICs), the majority of disease is caused by a limited number of *emm* types, with *emm*1 being the most common. A 30-valent M protein vaccine has been developed and is undergoing clinical trials but is based on genotypes circulating predominantly in Europe and North America (4, 5). The limited available data for S. pyogenes in LMICs suggest a far more genetically diverse population than that seen in HICs (6–9). More extensive, global genomic analysis may reveal another vaccine target or combination of targets that would be applicable in these settings. Additionally, it provides a detailed molecular basis for pathogenesis studies that are essential to our understanding of S. pyogenes infection mechanisms as well as vaccine and new therapy research.

The *emm* gene lies within the S. pyogenes core *mga* (multigene activator) regulon locus, and upstream and/or downstream of the *emm* gene there may be additional *emm-*like genes. There have been 10 different *emm* patterns identified, based on the genes within the *mga* regulon, which form three main groupings: A-C, D, or E. For the majority, each *emm* type has been associated with a single *emm* pattern (10). There is epidemiological evidence supporting the existence of tissue tropism among *emm* types, with preference for either pharyngeal or skin infection sites or “generalists” that are equally able to infect both sites (11). There is also an association with this tissue tropism to *emm* pattern: pharyngeal specialists are pattern A-C, the skin specialists are pattern D, and the generalists are pattern E (10). However, much of this evidence comes from population-based surveys where there is greater sampling of pharyngeal infections in HICs but more skin infections (impetigo/pyoderma) in LMICs (12). Whether this reflects a true difference in the prevalence of infection types is unclear, as data are lacking for both skin infections in HICs and pharyngeal infections in LMICs (12–14).

It is estimated that more than 162 million children have impetigo/pyoderma at any given time, predominantly in LMICs, although data for Europe, South East Asia, and North America are limited (14). A recent study in The Gambia, West Africa, identified a 17.4% prevalence of pyoderma in children, with S. pyogenes as a leading infection cause (15). This was higher than the estimated global prevalence of 12.3% (14). The association with scabies infestation in The Gambia was lower than that seen in other settings, but there was an increase in pyoderma prevalence from 8.9% to 23.1% during the rainy season (15). While environmental and sociodemographic factors may underpin the high burden of pyoderma in The Gambia, bacterial factors influencing epidemiology and tissue tropism may also have a role. To investigate this, and to provide molecular characterization of S. pyogenes causing skin infections in The Gambia, we performed whole-genome sequencing (WGS) on the isolates obtained from our previous study (15). We also performed WGS and molecular characterization of S. pyogenes isolated from skin infections in Sheffield, United Kingdom, as a comparative HIC collection of isolates. In addition, we compared our isolate genomes to publicly available genomes from collections representing HIC and low-income country (LIC) isolates. As our isolates represented skin infections, variable genomic loci thought to contribute to skin infection tropism were also analyzed. We also looked for other bacterial factors that might be linked to geographical origin.

## RESULTS

### The *emm* type diversity of LIC and HIC S. pyogenes isolates.

We performed WGS on 115 S. pyogenes skin infection isolates collected in The Gambia (15). After quality control and filtering of reads and *de novo* assemblies, we obtained high-quality genome sequence data for a total of 107 Gambian (LIC) S. pyogenes isolates for further analyses (see Table S1 in the supplemental material). Within the genomes of these 107 isolates, we determined 46 different *emm* types, with no obvious dominant *emm* type, the most common being *emm*80 (6/107, ~6%), closely followed by *emm*85, *emm*229, and *emm/*stG1750 (5/107 isolates, ~5% each) (Fig. S1A). Although *emm*/stG1750 has been previously identified in group G streptococci, in this case these isolates were S. pyogenes with the group A carbohydrate. The Simpson’s reciprocal index (SRI) was 49.3 (95% confidence interval [CI], 39.0 to 66.9), indicating high *emm* type diversity and similar to 41.6, which was previously calculated for S. pyogenes isolates in The Gambia collected between 2004 and 2018 (9). The multilocus sequence types (STs) for all 107 isolates were determined and revealed 57 different types, of which 25 were assigned for the first time (Table S1). Although multiple STs could be found within single *emm* types, no STs were shared by multiple *emm* types.

10.1128/msphere.00469-22.1 FIG S1 Distribution of *emm* types and pairwise SNP distances. (A) Frequency of each of the 62 *emm* types identified in the Gambian LIC isolates (black) and the Sheffield HIC isolates (white). Highlighted on the *x* axis are *emm* types found only in LIC (Gambia, Kenya, and Fiji; black circles) isolates or only in HIC (BSAC, PHE, ABCs, and Sheffield; white circles) isolates. (B and C) SNPs were determined from the core genome of 107 Gambian LIC isolates (B) and 142 Sheffield HIC isolates (C), and pairwise distance was calculated between isolates belonging to the same (red) or different (blue) *emm* type. Overall, the median pairwise SNP distance within the same *emm* type of LIC isolates was 20 (range, 0 to 13,914 SNPs), which was similar to that of the Sheffield HIC isolates with a median of 20 (range, 0 to 2,677). Also comparable was the between-*emm-*type median SNP distances of 12,379 (range, 1,649 to 17,173) for the Gambian LIC isolates and 13,186 (range, 3,808 to 15,476) for the HIC isolates. Download FIG S1, TIF file, 2.0 MB. Copyright © 2022 Bah et al. 2022 Bah et al. https://creativecommons.org/licenses/by/4.0/ This content is distributed under the terms of the Creative Commons Attribution 4.0 International license.

10.1128/msphere.00469-22.9 TABLE S1 Isolate metadata and summary of genome sequence findings. Download Table S1, XLSX file, 0.1 MB. Copyright © 2022 Bah et al. 2022 Bah et al. https://creativecommons.org/licenses/by/4.0/ This content is distributed under the terms of the Creative Commons Attribution 4.0 International license.

To act as a comparison HIC population for the skin infection isolates, we also obtained draft genomes after read quality filtering and assembly assessment for 142 S. pyogenes isolates collected in Sheffield, United Kingdom. Within these 142 Sheffield HIC isolates, there were 23 different *emm* types but ~59% of the isolates were represented by just 5 *emm* types: *emm*108 (30/142, 21%), *emm*89 (19/142, 13%), *emm*12 (15/142, 11%), *emm*1 (10/142, 7%), and *emm*4 (9/142, 6%) (Fig. S1A). The SRI was calculated at 11.1 (95% CI, 8.8 to 14.9), indicating far lower diversity than that of the Gambian LIC isolates. Consistent with the fewer *emm* types within the Sheffield HIC isolate collection, we identified only 28 different STs, the most common being ST14, ST101, ST36, and ST28, reflective of their association with the dominant *emm* types *emm*108, *emm*89, *emm*12, and *emm*1, respectively. As with the Gambian LIC isolates, STs were unique to a single *emm* type (Table S1). Surprisingly, only 7 out of the 62 total *emm* types identified were common to both the Gambian LIC and the Sheffield HIC isolates: *emm*4, -28, -75, -77, -80, -81, and -89. However, except for *emm*80 (*emm*80.0), they were different *emm* subtypes between the two sites (Table S1).

To provide further comparative data and to determine if our collection of Gambian LIC isolates and Sheffield HIC isolates were representative of LIC and HIC isolates in general, we obtained publicly available data for well characterized and previously assembled and curated S. pyogenes genome collections. For the wider LIC isolate genome collection, we used data for 328 isolates from Kenya (7, 16) and 346 isolates from Fiji (16, 17). These isolates were from a wide range of infections that were predominantly invasive for Kenyan isolates and a mixture of invasive and noninvasive for Fijian isolates that included both throat and skin infections (7, 16, 17). For the wider HIC isolate genome collection, we used data for 344 BSAC (British Society for Antimicrobial Chemotherapy) isolates from across England/Wales (18), 2,898 PHE (Public Health England) isolates collected from across the United Kingdom (19), and 1,436 ABCs (Active Bacterial Core surveillance) isolates collected across the United States (20). The BSAC isolates and ABCs isolates were from invasive disease, and although the clinical origin for PHE isolates is not known, the majority were likely to have been isolated from invasive infections and outbreak situations (19).

For the wider LIC isolate genome collection, there was a high number of different *emm* types identified with no dominant *emm* type: 88 *emm* types in Kenya with *emm*44 (~5%), *emm*65 (~4%), and *emm*/STG866 (~4%) most common and 59 *emm* types in Fiji with *emm*25 (~8%), *emm*11 (~6%), and *emm*93 (~5%) most common. The calculated SRIs were 65.6 (95% CI, 56.2 to 78.4) and 32.8 (95% CI, 28.5 to 38.6) for Kenya and Fiji, respectively, indicating high diversity as with our Gambian LIC isolates. Forty-two of the 46 *emm* types found in our Gambian isolates were also found in Kenyan or Fijian LIC isolates.

**FIG 1:**
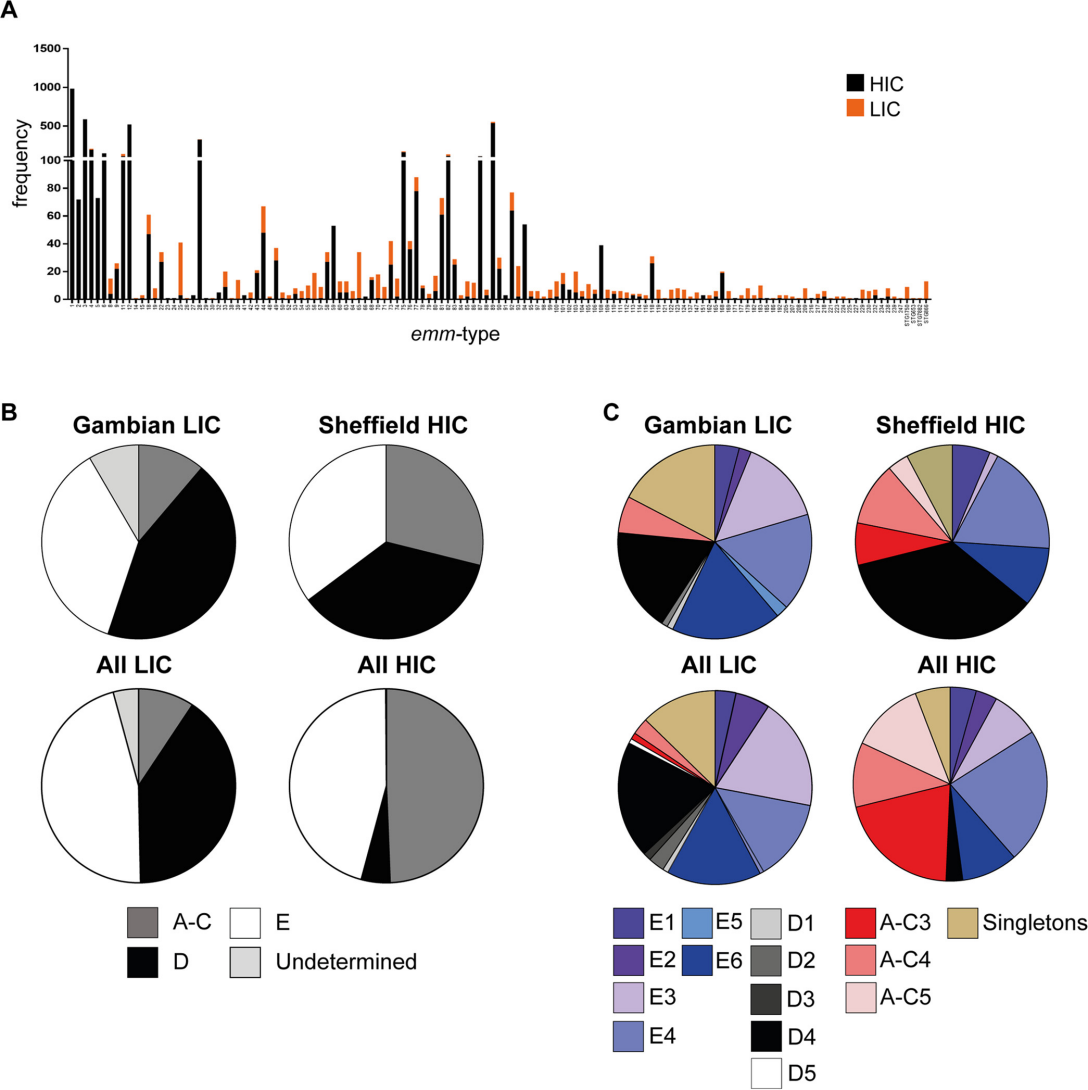
Distribution of *emm* type, pattern, and cluster differs by site. (A) Frequency of *emm* types found in the wider LIC genome collection and Gambian isolate genomes combined (LIC; orange) compared to the that found in the wider HIC genome collection and Sheffield isolate genomes combined (HIC; black) (B) An *emm* pattern of A-C, D, or E was assigned to 98/107 Gambian LIC isolates (patterns for the remaining 9 were undetermined) and all 142 Sheffield HIC isolates. The same *emm* patterns were also assigned to 766/781 (15 were undetermined) LIC isolates from Gambia, Kenya, and Fiji (All LIC) and all 4,820 HIC isolates from Sheffield, BSAC (England/Wales), PHE (United Kingdom), and ABCs (United States) (All HIC). (C) An *emm* cluster was also assigned to 98/107 Gambian LIC isolates (the remaining 9 were excluded) and all 142 Sheffield HIC isolates, as well as to all LIC and all HIC isolates. Pie charts represent the percentage of isolates associated with each pattern/cluster.

There were 92 different *emm* types within the 4,678 wider HIC isolate genomes, and in all three collections, *emm*1 was the most common, with *emm*12 and *emm*89 in the top four, alongside *emm*3 for UK isolates or *emm*82 for U.S. isolates. The SRI indicated low *emm* type diversity in each of these HIC collections compared to that calculated for the LIC collections: 13.9 (95% CI, 11.7 to 16.9) for BSAC, 9.1 (95% CI, 9.5 to 8.6) for PHE, and 10.8 (95% CI, 9.9 to 11.9) for ABCs. As we observed in our Sheffield HIC isolates alone, 61.3% of all the HIC isolates (*n* = 4,820) were represented by just five *emm* types: *emm*1 (20.4%), *emm*3 (12.2%), *emm*89 (11.2%), *emm*12 (10.8%), and *emm*28 (6.7%) (Fig. 1A). This was very different from all the LIC isolates (Gambia, Kenya, and Fiji, *n* = 781), where *emm*25 (4.9%), *emm*65 (4.2%), *emm*11 (3.6%), and *emm*93 (2.8%) were the most common, and 33 different *emm* types represented the equivalent of ~60% of the population (Fig. 1A).

Of the 62 *emm* types found in our Sheffield HIC and Gambian LIC isolates, 18 were found only in LIC isolates (Gambia, Kenya, and Fiji) and 9 were found only in HIC isolates (Sheffield, BSAC, PHE, and ABCs) (Fig. S1A). Across all the LIC and HIC isolate genome collections, there were 135 different *emm* types, of which 42 *emm* types were found only in LIC isolate genomes (Fig. 1A). Twenty-four of 135 were absent from all LIC isolate genomes. Although the majority were also rare in HIC isolate genomes (1 to 7 isolates for each type), 9 of these 24 LIC-absent *emm* types were found in 1 to 20% of HIC isolates and overall comprised 52% of all the HIC isolates and included *emm*1, *emm*2, *emm*3, *emm*5, *emm*6, and *emm*12.

### The *emm* patterns and clusters of LIC and HIC S. pyogenes isolates.

An *emm* pattern could be assigned to the majority of Gambian LIC isolates and all Sheffield HIC isolates by using the previously determined classifications. The exceptions were two Gambian LIC *emm*147 isolates, one Gambian LIC *emm*162 isolate, one Gambian LIC *emm*247 isolate, and five Gambian LIC *emm*/stG1750 isolates, for which an *emm* pattern had not been previously described. Of the 98 Gambian LIC isolates with known *emm* patterns, 48% (*n* = 47) were D (skin associated), 40% (*n* = 39) were E (generalists), and 12% (*n* = 12) were A-C (throat associated) (Fig. 1B). A similar distribution of *emm* patterns was also found in all LIC isolates (Gambia, Kenya, and Fiji, *n* = 781), with the majority being D (40%) or E (46%) and with very few A-C (9%).

The *emm* pattern distribution in our 142 Sheffield HIC isolates was 36% (*n* = 51) D, 35% (*n* = 50) E, and 29% (*n* = 41) A-C (Fig. 1B). For all HIC isolates (Sheffield, BSAC, PHE, and ABCs, *n* = 4,820), 5% were D, 45% were E, and 49% were A-C. The difference in distribution may again be due to the different types of infections caused by the isolates in the wider HIC isolate genome collection, as they represented high levels of invasive infections compared to our Sheffield skin infection isolates. The upsurge of *emm*108 at the time of collection also contributed to the high number of D pattern isolates in our Sheffield collection but was still higher, at 19%, when *emm*108 was excluded.

An *emm* cluster type could also be assigned to the isolates for which an *emm* pattern had been assigned (Fig. 1C). The *emm* cluster type is based on the sequence of the full M protein and is broadly associated with *emm* pattern (21). The majority of the Gambian LIC isolates (56/98, ~57%) were assigned to one of the six E *emm* cluster types: E1 (*n* = 4), E2 (*n* = 2), E3 (*n* = 14), E4 (*n* = 16), E5 (*n* = 2), and E6 (*n* = 18), representing 25 *emm* types (Fig. 1C). All E1 to E4 and all but four E6 *emm* types were positive for the serum opacity factor (*sof*) gene, commonly associated with E *emm* clusters (11); however, E5 *emm* types were *sof* negative. The remaining isolates were A-C4 (*n* = 6), D1 (*n* = 1), D2 (*n* = 1), D4 (*n* = 17), or singletons (*n* = 17). The majority of the Sheffield HIC isolates were D4 (*n* = 50, 35%). No other D cluster types were found. The most common E cluster type was E4 (*n* = 26), followed by E6 (*n* = 14), E1 (*n* = 9), and E3 (*n* = 2). The A-C clusters were represented by *emm*1 (A-C3, *n* = 10), *emm*12 (A-C4, *n* = 15), and *emm*3 (A-C5, *n* = 5), which were *emm* types absent from the Gambian LIC population. Only *emm*5 (*n* = 4) and *emm*6 (*n* = 7) were singleton *emm* cluster types.

Very similar *emm* cluster distributions were identified for all LIC and all HIC isolate genomes compared to those for Gambian LIC and Sheffield HIC isolate genomes, respectively (Fig. 1C), although a higher proportion of all HIC isolates were A-C3 and fewer were D4 than were Sheffield HIC isolates alone.

### Core genome phylogeny of LIC and HIC S. pyogenes isolates.

**FIG 2:**
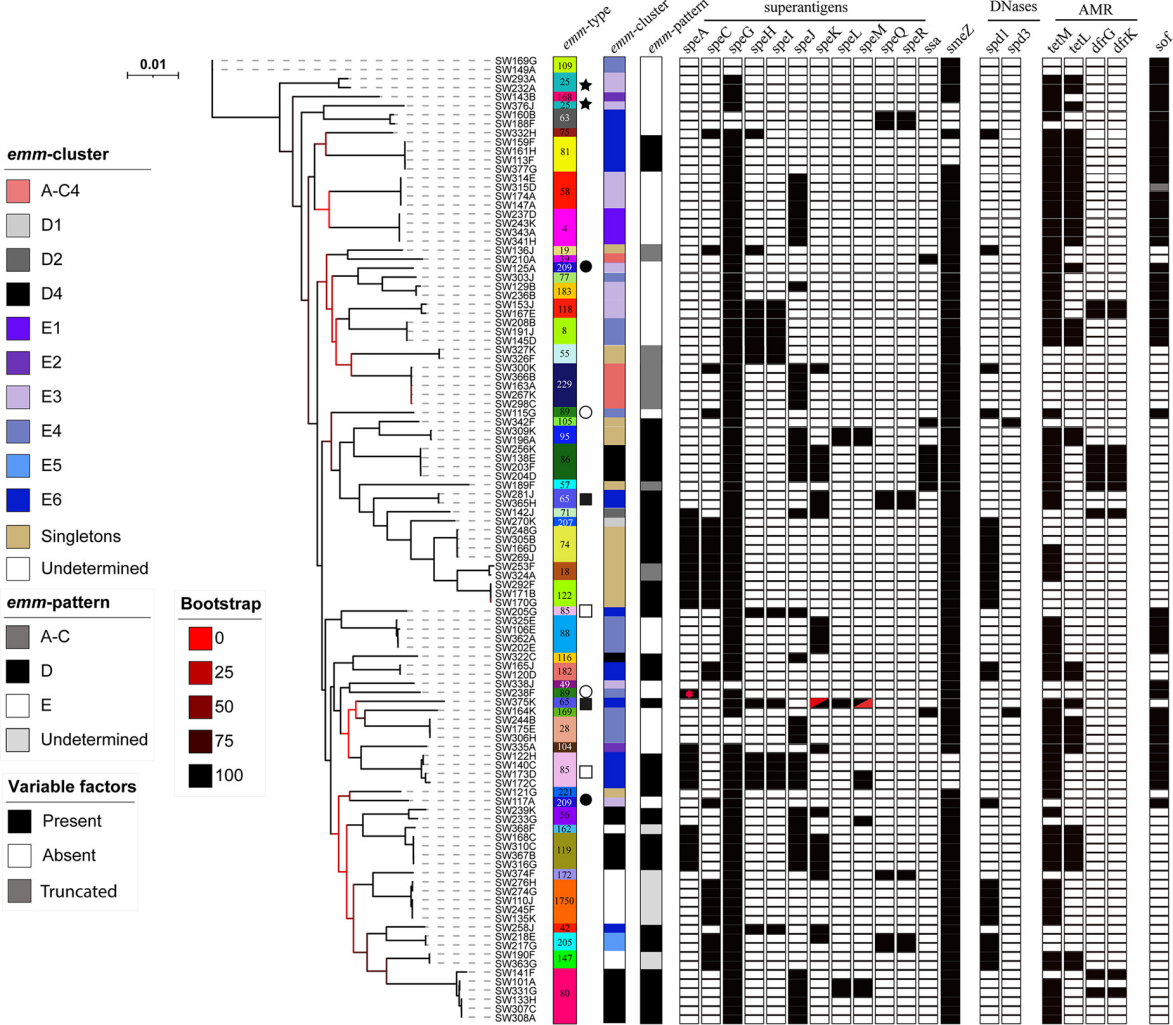
Phylogenetic analysis of the 107 Gambian LIC isolate genomes. A maximum likelihood phylogeny was constructed from the core gene alignment (1,382,412 bp) using RAxML (51) with 100 bootstraps. Isolates clustered by *emm* type except for those indicated, whereby two lineages were represented by a single *emm* genotype: star, *emm*25; filled square, *emm*65; open square, *emm*85; open circle, *emm*89; filled circle, *emm*209. Also shown is the presence (black)/absence (white) of the superantigen genes *speA*, *speC*, *speG*, *speH* to *speM*, *speQ*, *speR*, *ssa*, and *smeZ* and DNase genes *spd1* and *spd3*; four other DNase genes (*sda1*, *sda2*, *sdn*, and *spd4*) were tested for but were not found in any isolate. In all cases except one (red dot), *speA* was located within the prophage-like element Φ10394.2. One isolate had a gene that appeared to be a fusion of 5′ *speK* and 3′ *speM* (red triangles). Antimicrobial resistance genes (AMR) *tetM*, *tetL*, *dfrG*, and *dfrK* were also identified in some isolates (white, absent; black, present). The positivity for serum opacity factor (*sof*) is also shown, although for one *emm*55 isolate, this gene would produce a truncated variant of serum opacity factor (gray). The scale bar represents substitutions per site. *emm* types are colored for easy visualization, and type numbers are also given.

**FIG 3:**
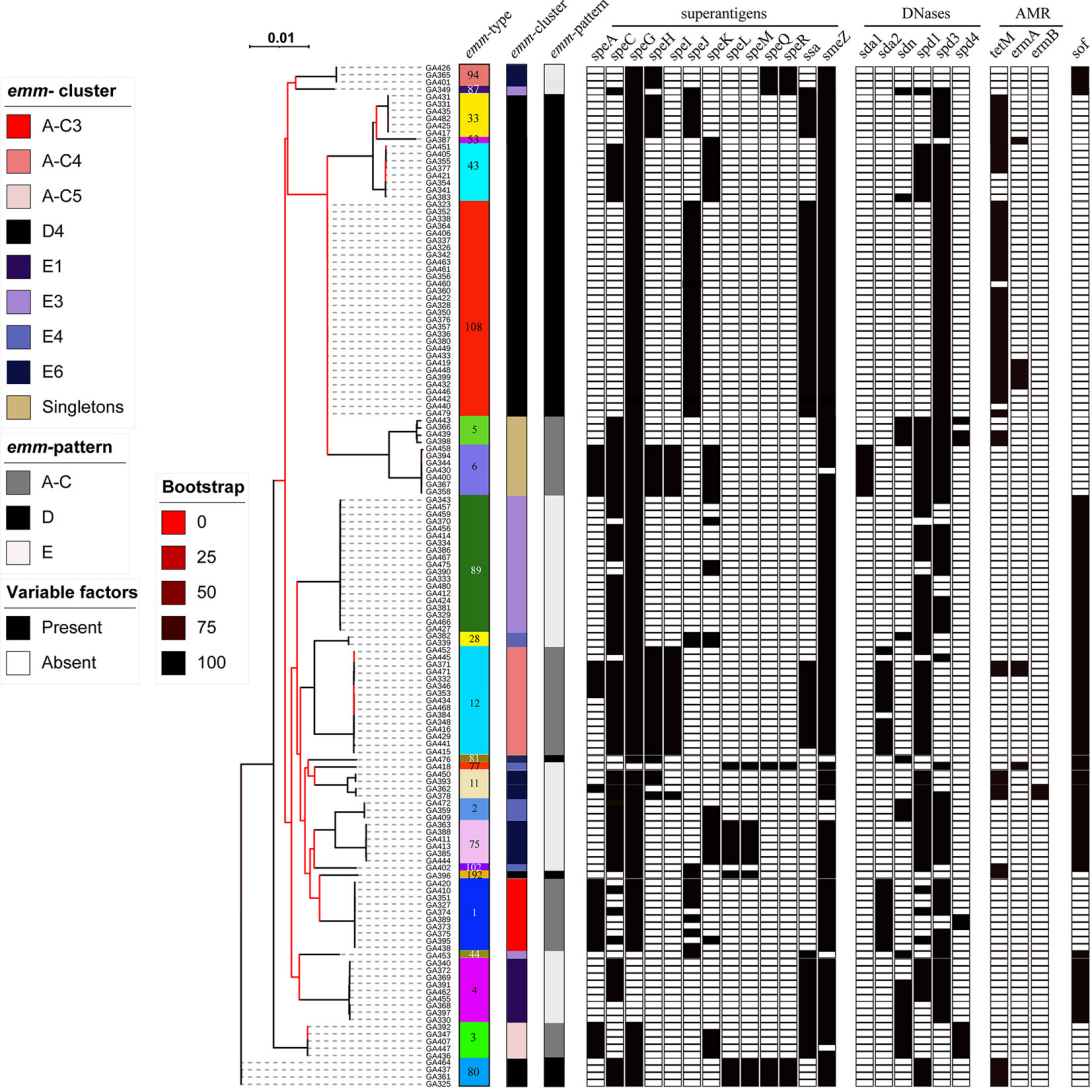
Phylogenetic analysis of the 142 Sheffield HIC isolate genomes. A maximum likelihood phylogenetic tree was generated with the core gene alignment (1,185,813 bp) by using RAxML (51) with 100 bootstraps. All isolates clustered by *emm* type. The presence (black)/absence (white) of superantigen genes *speA*, *speC*, *speG*, *speH* to *speM*, *speQ*, *speR*, *ssa*, and *smeZ* and DNase genes *sda1*, *sda2*, *sdn*, *spd1*, *spd3*, and *spd4* is indicated. Antimicrobial resistance genes (AMR) *tetM*, *ermA*, and *ermB* were also identified in some isolates (white, absent; black, present). The positivity for serum opacity factor (*sof*) is also shown, but in all *emm*12 types, this gene would produce a truncated variation of serum opacity factor (gray). The scale bar represents substitutions per site. *emm* types are colored for easy visualization, and type numbers are also given.

Phylogenetic analysis of the core genome of all 107 Gambian LIC isolates showed clustering by *emm* type (Fig. 2). The exceptions to this were *emm*25, *emm*65, *emm*85, *emm*89, and *emm*209, whereby two distinct core genome lineages were identified within these genotypes. The phylogenetic analysis of the Sheffield HIC isolates based on core genome single nucleotide polymorphisms (SNPs) also grouped isolates into lineages based on *emm* types, and all *emm* types formed single lineages (Fig. 3).

Pairwise distance analysis of the core genome alignment of all Gambian LIC and Sheffield HIC isolates combined identified a median of 20 SNPs when Gambian LIC isolates of the same *emm* type were compared (range, 0 to 13,914 SNPs) and a median of 12,379 SNPs when Gambian LIC isolates with different *emm* types were compared (range, 1,649 to 17,173 SNPs) (Fig. S1B). Excluding isolates of the *emm* types demonstrating two distinct phylogenetic lineages reduced the range of intra-*emm* types to 0 to 1,805 SNPs. Pairwise genetic distance analysis between Sheffield HIC isolates also identified a median of 20 SNPs between isolates of the same *emm* type (range, 0 to 2,677), compared to a median distance of 13,186 SNPs between isolates of different *emm* types (range, 3,808 to 15,476) (Fig. S1C). Sheffield HIC *emm*11 isolates demonstrated the highest level of intra-*emm* type diversity, ranging 1 to 2,677 SNPs. Excluding this *emm* type reduced the pairwise genetic distance range to 0 to 291 SNPs, suggesting lower genetic diversity in the Sheffield HIC isolates of the same *emm* type than in the Gambian LIC isolates.

Pairwise comparison of isolates from the two different sites where the *emm* type was shared (*emm*4, -28, -75, -77, -80, -81, and -89) revealed a level of genetic distance similar to that observed when isolates of different *emm* types were compared (median, 13,462 SNPs; range, 12,420 to 14,927 SNPs), indicating that although they may share an *emm* type, they do not share a core genome. There was also no core genome phylogenetic clustering of the Sheffield HIC isolates with the Gambian LIC isolates of the same *emm* type (Fig. S2).

10.1128/msphere.00469-22.2 FIG S2 Combined population structure of Gambian LIC (107) and Sheffield HIC (142) isolates. A maximum likelihood phylogenetic was generated from the core gene alignment (1,360,768 bp) using RAxML (51) with 100 bootstraps. Bootstrap support is indicated by the colors in the legend. Outer circle, site of collection; middle circle, ST; inner circle, *emm* type. The ST and *emm* type numbers are provided for each terminal branch. Highlighted are the lineages for the *emm* types shared between Sheffield HIC and Gambian LIC isolates, and they all differ extensively by core genome: *emm*4, *emm*28, *emm*75, *emm*77, *emm*80, *emm*81, and *emm*89. There are two lineages for Gambian LIC *emm*89, but neither one clusters with Sheffield HIC *emm*89. The tree was midpoint rooted. The scale represents substitutions per site. Download FIG S2, TIF file, 2.8 MB. Copyright © 2022 Bah et al. 2022 Bah et al. https://creativecommons.org/licenses/by/4.0/ This content is distributed under the terms of the Creative Commons Attribution 4.0 International license.

It is also possible that closely related isolates may exist within both collections but carry different *emm* genes. Core gene phylogeny of all isolates from the two geographical sites combined showed clear segregation of isolates from different sites, except in one instance where an *emm*192 Sheffield HIC isolate clustered with two *emm*56 Gambian LIC isolates (Fig. S2).

To place our Gambian and Sheffield isolates into a wider genomic context, we subsampled the LIC and HIC isolate genome collections to include at least 10 isolate genomes from each geographical site and of the same ST for each of the 62 Sheffield/Gambian *emm* types. Pangenome analysis was performed on the 1,368 subsampled genomes, and a phylogenetic tree was constructed from the core genome alignment (Fig. S3). When a pairwise comparison was made of the core genome alignment of isolates of the same *emm* type where that *emm* type was identified only in HIC isolates (*n* = 9 *emm* types), the median genetic distance was 65 SNPs with a range of 0 to 15,075 SNPs. The greatest distance was between the *emm*102 isolates, as this *emm* type exists as two very separate lineages; exclusion of this *emm* type gave a range of 0 to 807 SNPs. This indicates that our Sheffield isolates are genetically similar to other HIC isolates of the same *emm* type, and this was also shown phylogenetically (Fig. S3).

10.1128/msphere.00469-22.3 FIG S3 Phylogeny of a subsample of the wider LIC and HIC isolate genomes. A core genome alignment was generated by subsampling for Panaroo analysis at least 10 isolate genomes for each of the 65 *emm* types found in Sheffield and/or Gambian isolates within each collection (Sheffield, The Gambia, PHE, BSAC, ABCs, Kenya, or Fiji) with the same ST. Where ST differed within an *emm* type, these isolate genomes were additionally included. The core genes (*n* = 1,366) of the 1,368 genomes were aligned and used to generate the maximum likelihood phylogenetic tree using RAxML with 100 bootstraps (scale shown). Inner ring, isolate genome collection; middle ring, LIC (orange) or HIC (black) isolates; outer ring, *emm* type (color code and number provided). Of the 65 *emm* types identified in the Sheffield and Gambian isolates, 35 *emm* types were found in both HIC and LIC isolates over the wider collections. Those commonly found in HIC isolates are highlighted around the outside of the tree with indication of whether they formed LIC-only (orange circles), HIC-only (black circles), or HIC-and-LIC-combined (orange and black circles) lineages: *emm*4, *emm*11, *emm*18, *emm*28, *emm*44, *emm*75, *emm*77, *emm*81, *emm*87, and *emm*89. Also highlighted are the lineages of the most common Sheffield HIC *emm* types (*emm*1, *emm*3, *emm*12, and *emm*108; within black boxes) and the most common Gambian *emm* types (*emm*80, *emm*85, and *emm/*stG1750; within oranges boxes), also with an indication of whether they are LIC-only, HIC-only, or HIC-and-LIC-combined lineages. Download FIG S3, TIF file, 2.1 MB. Copyright © 2022 Bah et al. 2022 Bah et al. https://creativecommons.org/licenses/by/4.0/ This content is distributed under the terms of the Creative Commons Attribution 4.0 International license.

Inclusion of the wider LIC and HIC isolate genome collections identified 35 out of the 62 total Sheffield/Gambian *emm* types in both LIC and HIC isolates. These 35 LIC and HIC shared *emm* types included those commonly found (>1% of isolates) in HIC isolates: *emm*89 (11.2%), *emm*28 (6.7%), *emm*4 (4.0%), *emm*75 (3.3%), *emm*11 (2.3%), *emm*87 (2.2%), *emm*77 (1.6%), *emm*81 (1.3%), *emm*44 (1.0%), and *emm*18 (1.0%). For *emm*28 and *emm*89 isolates, although both formed several different lineages, there were separate HIC lineages and LIC lineages with no overlap (Fig. S3). Multiple lineages (2 to 5) also existed within *emm*4, *emm*18, *emm*44, *emm*75, *emm*77, and *emm*81 isolates, but for each *emm* type, one lineage, or two in the case of *emm*18, contained both HIC and LIC isolates. For *emm*11, there was a distinct LIC isolate lineage as well as a separate cluster formed of both HIC and LIC isolates, although within this cluster were sublineages divided by geographical location. For *emm*87, there was a single HIC isolate lineage, and within this was an LIC isolate (Fijian) sublineage. There was also some clustering of HIC and LIC isolates within the *emm* types common to The Gambia, *emm*80 and *emm*85 (Fig. S3), although *emm*229 and *emm/*stG1750 were LIC-only lineages.

### Geographical site-associated genes identified by pangenome analysis.

The core genome of isolates from Sheffield and The Gambia combined was estimated to be 1,358 genes from a total of 3,804 genes. To identify genes that may be overrepresented in or unique to isolates from one geographical site compared to the other, genes found in 10% or fewer of all isolates were excluded, along with those found in 90 to 100% of all isolates. This left 798 genes for which 117 were unique to one or more Sheffield HIC isolates and 65 were unique to one or more Gambian LIC isolates. All 117 Sheffield HIC-unique genes were predicted to be prophage/transposon related.

Of the 65 Gambian LIC-unique genes, 38 were predicted to be associated with prophage/transposases or antimicrobial resistance loci, and for 17 genes, no function could be predicted. The remaining 10 Gambian LIC-unique genes were found within a single operon previously identified to be involved in the production of the streptococcin A-FF22 lantibiotic (22) (Table 1). A total of 38 (36%) of Gambian LIC isolates carried this operon, all with the two *scnA* genes *scnA-*1 and *scnA-*3, but 19 also carried *scnA-*2 (Table S1). All three genes encode a 51-amino-acid (51-aa) peptide. The ScnA-2 peptide was previously identified as streptococcin A-M49 (23) and differs from ScnA-1 (streptococcin A-FF22) by just 4 amino acids. The *scnA*-3 gene was previously termed streptococcin A-FF22′ but differs substantially from both *scnA-*1 and *scnA*-2 (22). The streptococcin operon lies within a variable region of the streptococcal chromosome, between the equivalent H293 reference genes SPYH293_00487 (*mefE*) and SPYH293_00499 (haloacid dehalogenase-like [HAD] superfamily hydrolase). All Sheffield HIC and Gambian LIC isolates, and the reference genome H293, carried a toxin-antitoxin locus (*relB/relE*) within this region as well as 8 or 9 genes that are prophage/transposon related. In addition, 80 (75%) Gambian LIC isolates and 43 (30%) Sheffield HIC isolates had an additional ~6 to 9 phage/transposon-related genes and a gene predicted to encode a microcin C7 self-immunity protein, MccF (Table S1). The number of phage/transposon-related genes varied, as they were truncated or misannotated in some isolate genomes (Fig. S4). Of the 38 Gambian LIC isolates that carried the streptococcin operon within this region, 33 also carried the *mccF* gene and the additional phage/transposon-related genes. We also determined the presence of the streptococcin operon in the wider LIC and HIC isolate genomes and found that 28% of all LIC isolate genomes carried the operon compared to just 1% of all HIC isolate genomes (Table 1). The presence of the operon was slightly higher in Kenyan isolate genomes (42%) than in Gambian isolate genomes (36%) but was much lower in Fijian isolate genomes (13%).

**TABLE 1:** Differentially prevalent operons by geographical site

Operon	Gene(s)	Predicted function	No. (%) of Gambian LIC isolates	No. (%) of Sheffield HIC isolates	No. (%) of all LIC isolates^*a*^	No. (%) of all HIC isolates^*b*^
Streptococcin A-FF22 lantibiotic	*scnK, scnR*	Response histidine kinase, regulator	38 (36)	0 (0)		
	*scnA1*	Lantibiotic	38 (36)	0 (0)	222 (28)	59 (1)
	*scnA2*	Lantibiotic	19 (18)	0 (0)		
	*scnA3*	Lantibiotic	38 (36)	0 (0)		
	*scnM*	Modifying enzyme	38 (36)	0 (0)		
	*scnT*	Transport and leader peptide cleaver	38 (36)	0 (0)		
	*scnF, scnE, scnG*	Immunity operon	38 (36)	0 (0)		
						
Glucuronic acid (GCA) utilization	*uidA*	Beta-glucuronidase	51 (48)	15 (11)	332 (42)	581 (12)
	*uxaC, uxuB, uxuA, kdgK, kdgA*	GCA utilization pathway	51 (48)	15 (11)		
	*yagG*	Sugar transporter	51 (48)	15 (11)		
	*fadR*	Regulator	51 (48)	15 (11)		
	*hypothetical gene*	Hydrolase	51 (48)	15 (11)		
						
CRISPR	*cas3, cas5c, cas8c, cas7, cas4, cas1, cas2*	Type 1C	54 (50)	74 (52)	315 (40)	3,471 (72)
	*cas9, cas1, cas2, csn2*	Type 2A	41 (38)	104 (73)	310 (39)	3,687 (76)

a All LIC isolates: Gambia, Kenya, and Fiji (*n* = 781) isolates.

b All HIC isolates: Sheffield, BSAC (England/Wales), PHE (United Kingdom), and ABCs (United States) isolates (*n* = 4,820). Only the *scnA* and *uidA* genes were used to determine the presence or absence of these loci in all the LIC or all the HIC isolate genomes.

10.1128/msphere.00469-22.4 FIG S4 Representation of the genomic region associated with the streptococcin operon. Between the reference genome H293 genes SPYH293_00486 and SPYH293_00487 (two gray arrows on the left), and SPYH293_00499 and SPYH293_00501 (three gray arrows on the right) was a region identified to differ between isolates. In the H293 genome were 9 prophage-related genes (orange) and a toxin-antitoxin locus (*relB/relE*; green). This region was shared by other isolates, but in some isolate genomes, represented by the reference genome MGAS10750 (accession no. CP000262), were additional prophage-related genes (orange), transposon-related genes (yellow), and the *mccF* (blue) gene, encoding a predicted microcin self-immunity protein. Nineteen LIC isolate genomes also carried the nine-gene streptococcin operon (red), as the reference genome *emm*58 (CP035443) represents, with *scnA-*1 and *scnA-*3. A further 19 LIC isolate genomes carried the *scnA-*2 gene as well as the other nine streptococcin operon genes, represented by the reference genome NCTC13737 (LS483425). The numbers of prophage/transposon genes differed between isolates due to truncations and misannotation, and therefore the reference genomes were used as representations of genomes with or without the streptococcin operon and/or the *mccF* gene. The figure was drawn using Easyfig. Download FIG S4, TIF file, 1.4 MB. Copyright © 2022 Bah et al. 2022 Bah et al. https://creativecommons.org/licenses/by/4.0/ This content is distributed under the terms of the Creative Commons Attribution 4.0 International license.

In addition to geographical region-unique genes, two gene clusters were found to be more common in isolates from one country than the other. One of these gene clusters was ~4 times more prevalent in Gambian LIC isolates than in Sheffield HIC isolates and was predicted to be involved in glucuronic acid (GCA) utilization (Table 1; Fig. S5). This cluster of ~14 or 15 genes was found in 51 (48%) Gambian LIC isolate genomes but only 15 (11%) Sheffield HIC isolates (Table S1). It was also found in 42% of all LIC isolate genomes compared to 12% of all HIC isolate genomes (Table 1). One gene (*uidA*) was predicted to encode a beta-glucuronidase that can remove terminal GCA from glycosaminoglycans (24). GCA can then enter a utilization pathway to produce glyceraldehyde 3-phosphate and pyruvate (Fig. S5). Other genes within the cluster were predicted to encode the proteins essential for the GCA utilization pathway and sugar transport (Table 1). There were also several genes with predicted beta-*N*-acetylglucosaminidase domains, but these varied in number and length, suggesting that they may be pseudogenes in some isolate genomes. Three isolates also had pseudogenes for *kdgK* or *uxaC*, and one was missing *uxuB* (Table S1), indicating that the GCA utilization pathway may not be functional in all isolates.

10.1128/msphere.00469-22.5 FIG S5 Schematic of a gene cluster predicted to be involved in the glucuronic acid utilization pathway. (A) A cluster of ~15 genes was identified in 51 Gambian LIC isolate genomes and 15 Sheffield HIC isolate genomes. The gene *yagG* was predicted to encode a sugar transporter and may be involved in importing disaccharides. The gene *uidA* was predicted to encode a beta-glucuronidase, capable of releasing glucuronic acid from glycosaminoglycans. The gene *fadR* was predicted to encode a gene regulator and may control the expression of genes within this operon. The gene *hyp* (hypothetical), downstream of *uxuB*, has no name but has functional domains related to HAD hydrolases. The gene *nag3* was predicted to encode a beta-*N*-acetylglucosaminidase but varied in size between isolates. The downstream genes, indicated by an asterisk, also were predicted to be related to beta-*N*-acetylglucosaminidases but appeared to be pseudogenes. It was not clear if any isolates would encode a functional beta-*N*-acetylglucosaminidase. The final gene in the operon, *hyp* (hypothetical), did not have any predicted functional domains. (B) The proteins encoded by the genes *uxaC, uxuB, uxuA, kdgK*, and *kdgA* were predicted to play roles in the glucuronic acid utilization pathway. Glucuronic acid, potentially released from hyaluronic acid by UidA, can be converted to fructuronic acid by the uronic isomerase UxaC. This is then converted to mannonate by the mannonic oxidoreductase UxuB. Mannonate is then converted to 2-keto-3-deoxygluconate (KDG) by the mannonic dehydratase UxuA. KDG is then phosphorylated to 2-keto-3-deoxy-6-phosphogluconate (KDGP) by the KDG kinase KdgK. KDGP is then converted to glyceraldehyde 3-phosphate and pyruvate by the KDGP aldolase KdgA. Glyceraldehyde 3-phosphate can then enter glycolysis. Download FIG S5, TIF file, 1.1 MB. Copyright © 2022 Bah et al. 2022 Bah et al. https://creativecommons.org/licenses/by/4.0/ This content is distributed under the terms of the Creative Commons Attribution 4.0 International license.

CRISPR locus genes were also found to be more prevalent in one population than the other. S. pyogenes can carry two CRISPR loci: type 2A and type 1C (25). The type 1C was almost equally common in the two populations (Table 1; Table S1). In contrast, type 2A region was found in only 38% of Gambian LIC isolates compared to 73% of Sheffield HIC isolates (Table 1; Table S1). The presence of the CRISPR loci corresponded with *emm* type lineages within each geographical site, except that only one of two Gambian LIC *emm*56 isolates carried CRISPR type 2A. When all LIC and all HIC isolate genomes were analyzed for the presence of CRISPR loci, the type 1C region was found in 40% of LIC isolate genomes and in 72% of HIC isolate genomes, higher than what was found in Sheffield isolate genomes alone. A similar pattern was observed for type 2A, where 39% of LIC isolates genomes were found to have this locus, but it was present in 76% of HIC isolate genomes (Table 1).

### Diversity of superantigens and DNases in skin isolates.

**FIG 4:**
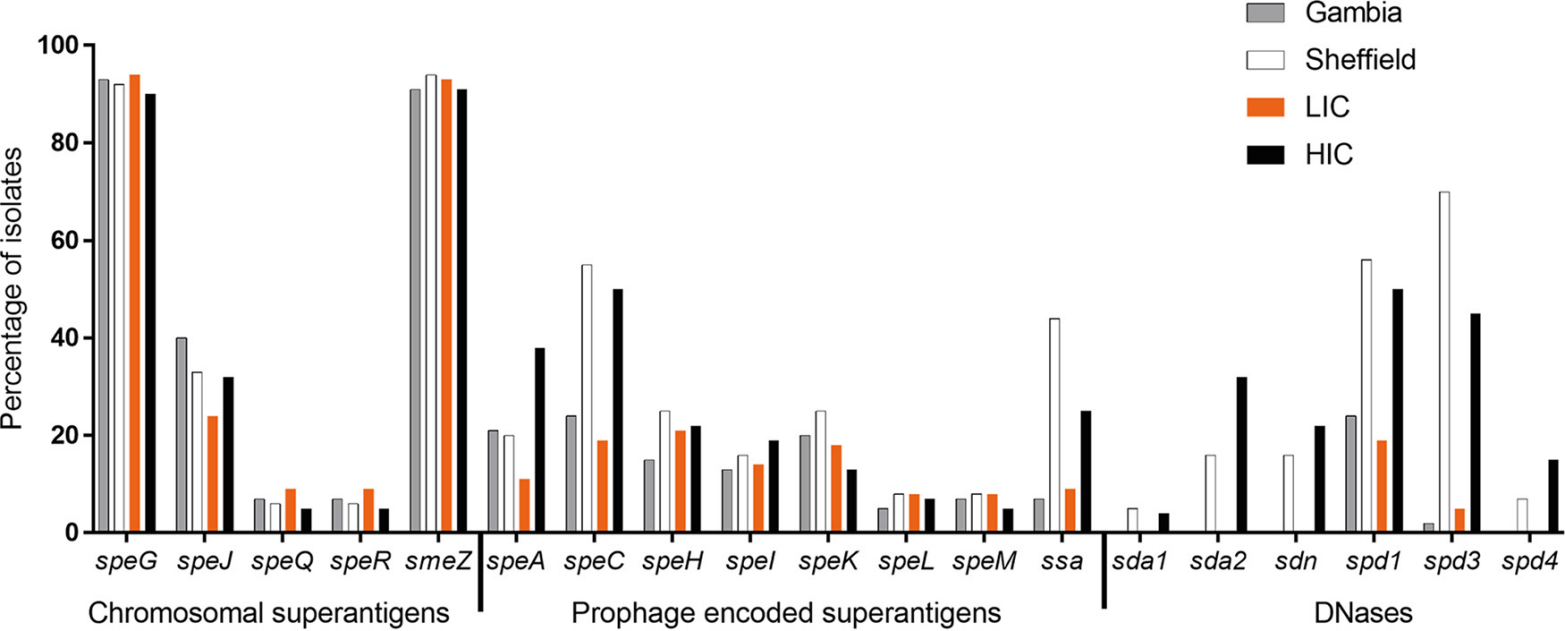
Comparison of superantigen and DNase gene carriage in LIC isolates and HIC isolates. The proportions of the Gambian LIC isolates (gray bars) and the Sheffield HIC isolates (white bars) as well as the wider LIC isolates (Kenya, Fiji, Gambia; *n* = 781) (orange bars) and the wider HIC isolates (BSAC, PHE, ABCs, Sheffield; *n* = 4,820) (black bars) carrying the respective genes were determined by BLAST analysis and mapping.

Pangenome analysis also identified variation between geographical sites in the prophage-associated and chromosomal superantigen and DNase genes. There are potentially 13 different superantigen genes that can be carried by S. pyogenes: *speA*, *speC*, *speH*, *speI*, *speK*, *speL*, *speM*, and *ssa* are prophage associated, while *speG*, *speJ*, *speQ*, *speR*, and *smeZ* are chromosomal. We tested for the presence of all known superantigen genes (results for each isolate are detailed in Table S1 and Table S2). Of the 107 Gambian LIC isolates, 99 (93%) carried *speG* and 97 (91%) had *smeZ*. Less common were *speJ* and the cotranscribed *speQ*/*speR*, found in 43/107 (40%) and 7/107 (7%), isolates, respectively (Fig. 4). A similar pattern was observed in the Sheffield HIC isolates, with 130/142 (92%) and 134/142 (94%) isolates carrying *speG* and *smeZ*, respectively, while *speJ* was present in 47/142 (33%) and *speQ/speR* was carried in 9/142 (6%) isolates (Fig. 4). A similar proportion of the wider LIC and wider HIC isolate genomes also carried *smeZ*, *speJ*, and *speQ/speR* (Fig. 4).

10.1128/msphere.00469-22.10 TABLE S2 Wider LIC and HIC isolate genome collection metadata. Download Table S2, XLSX file, 0.7 MB. Copyright © 2022 Bah et al. 2022 Bah et al. https://creativecommons.org/licenses/by/4.0/ This content is distributed under the terms of the Creative Commons Attribution 4.0 International license.

Of the prophage-associated superantigen genes, *speC* was the most predominant in the Gambian LIC isolates, carried by 26/107 (24%) isolates (Fig. 4), and also in the Sheffield HIC isolates, although much higher at 55% (78/142). Two (out of eight) *emm*43 Sheffield HIC isolates and the single *emm*102 Sheffield HIC isolate each carried two copies of *speC*, as well as two copies of the associated DNase gene *spd1*. These appeared to be carried on two separate phages integrated at two different sites. *speC* was also the most common prophage-associated superantigen gene found in the wider HIC isolate genomes.

In the Sheffield HIC isolates, prophage-associated *ssa* was present in 63/142 (44%) isolates, compared to only 8/107 (7%) of the Gambian LIC isolates. A similar proportion of the wider LIC isolate genomes also carried *ssa* (71/781, 9%), but fewer of the wider HIC isolate genomes than the Sheffield HIC isolate genomes (1,190/4,820, 25%) carried *ssa*.

Interestingly, *speA* was almost equally common in the Gambian LIC isolates (22/107, 21%) and the Sheffield HIC isolates (28/142, 20%), but apart from one *emm*89 isolate, all the Gambian LIC isolates carried the *speA4* allele (or *speA* alleles very close to this allele, encoding for *SpeA* variants that differ by 1 to 5 amino acids), which is 11% divergent from the other *speA* alleles (26) and was associated with a prophage-like element rather than a full prophage. This prophage-like element has been previously identified in the *emm*6 reference strain MGAS10394, termed Φ10394.2, and comprised transposase genes and fragments of *speH* and *speI* (Fig. S6A) (26). Previously, it had only been found in *emm*6, *emm*32, *emm*67, and *emm*77. In the Sheffield HIC isolate genomes, this element, and the *speA4* allele, was found only in *emm*6. The only isolate in the Gambian LIC isolate genomes that carried a different *speA* allele, one synonymous base pair different from *speA1*, was associated with a prophage, although this prophage did not share any substantial identity to other known prophages in S. pyogenes (determined by BLASTn search against the entire NCBI database). *speA* was more common (1,826/4,820, 38%) in the wider HIC isolate genomes than in LIC isolate genomes or Sheffield HIC isolate genomes alone, but only 9% of the *speA* genes detected in the wider HIC isolates were *speA4*. In comparison, 70% of the 89 *speA* alleles found in the wider LIC isolates were *speA4.*

10.1128/msphere.00469-22.6 FIG S6 (A) Comparison of Φ10394.2 phage-like element with the region found in HIC *emm*6 isolates and LIC isolates. The *speA4* in LIC isolates and HIC *emm*6 isolates were located within this phage-like element. This region also contains fragments of *speI* and *speH*. The same element was found in all Gambian LIC isolates that carried *speA*, except one that carried a different *speA* allele associated with a prophage. The corresponding regions were extracted from the respective isolate genomes, and the figure was generated using Easyfig. Red arrows, superantigen-associated genes; blue arrows, phage-like element genes; yellow arrows, chromosomal (nonelement) genes. (B) Alignment of the SpeK/SpeM fusion protein sequence to SpeK and SpeM. Within an *emm*65 isolate from LIC, we identified a gene predicted to encode 259 aa, of which the first 180 aa were 100% identical to the first 180 aa of SpeK (red underlined) but the remaining amino acids, aa 181 to 259, were 100% identical to the last amino acids, aa 159 to 237, of SpeM (blue underlined). Black shading indicates identical amino acids. Download FIG S6, TIF file, 2.4 MB. Copyright © 2022 Bah et al. 2022 Bah et al. https://creativecommons.org/licenses/by/4.0/ This content is distributed under the terms of the Creative Commons Attribution 4.0 International license.

Prophage-associated *speH*, *speI*, *speK*, *speL*, and *speM* were detected at fairly similar levels between the two sites, i.e., 15%, 13%, 20%, 5%, and 7%, respectively, in Gambian LIC isolates compared to 25%, 16%, 25%, 8%, and 8% in the Sheffield HIC isolates (Fig. 4). One Gambian LIC *emm*65 isolate had an apparent fusion gene comprised of 5′ *speK* and 3′ *speM*. An alignment of the 259 aa of the potential fusion protein showed 100% identity to the first 180 aa of SpeK and 100% of the remaining 181 to 259 aa to the last 159 to 237 aa of SpeM (Fig. S6B). This *speK/speM* gene was also identified in nine Fijian *emm*89 (ST380) isolates and one PHE *emm*89 (ST101) isolate, four Fijian *emm*100 isolates, five PHE *emm*49 isolates, and one PHE *emm*171 (Table S2).

We also tested for the presence of the prophage-associated DNase genes *sda*, *sdn*, *spd1*, *spd3*, and *spd4* (results for each isolate detailed in Table S1 and Table S2). Only two prophage-associated DNase genes were identified in the Gambian LIC isolates: *spd1* in 26/107 (24%) isolates and *spd3* in 2/107 (2%) isolates. These were also the most prevalent in the Sheffield HIC isolates, at 79/142 (56%) and 99/142 (70%), respectively, but we also detected *sda1* in 7/142 (5%), *sda2* in 23/147 (16%), *sdn* in 23/147 (16%), and *spd4* in 10/142 (7%) isolates. We also found the DNase genes to be rare in the wider LIC isolate genomes, as with the Gambian isolates alone: *sda1*, *sdn*, and *spd4* were entirely absent, and only one Kenyan isolate carried *sda*2. The *spd1* and *spd3* genes were detected but at much lower levels (19% and 5%, respectively) than in the wider HIC isolate genome collection (50% and 45%, respectively) (Fig. 4).

### The Mga regulon diversity.

The core Mga regulon includes the *mga* gene and all intervening genes up to and including *scpA* (encoding the C5a peptidase). Genes within this region encode proteins involved in cell invasion and immune evasion and include those for the M protein, encoded by *emm*, and the M-like proteins Mrp and Enn. The composition of the intervening genes that define the Mga regulon, as well as the type of M protein and positivity for serum opacity factor (*sof*), relates to the *emm* pattern (A-C, D, or E) and may be key to tissue tropism (10, 27). We therefore compared this region in our Gambian isolate genomes to that of our Sheffield isolate genomes as they represented skin infection isolates. We were able to determine the composition of the Mga regulon for 36/46 *emm* types for 85/107 Gambian LIC isolates and all 23/23 *emm* types for 139/142 Sheffield HIC isolates. Among the Gambian LIC isolates, we could not confirm the Mga regulon for all isolates within 10 different *emm* types because it was not contiguous in the *de novo* assemblies, possibly due to sequence quality or repetitive regions. For the Sheffield HIC isolates, this was the case for only single isolates within *emm* types *emm*1, *emm*12, and *emm*108, and other isolates within these *emm* types had confirmed Mga regulons.

**FIG 5:**
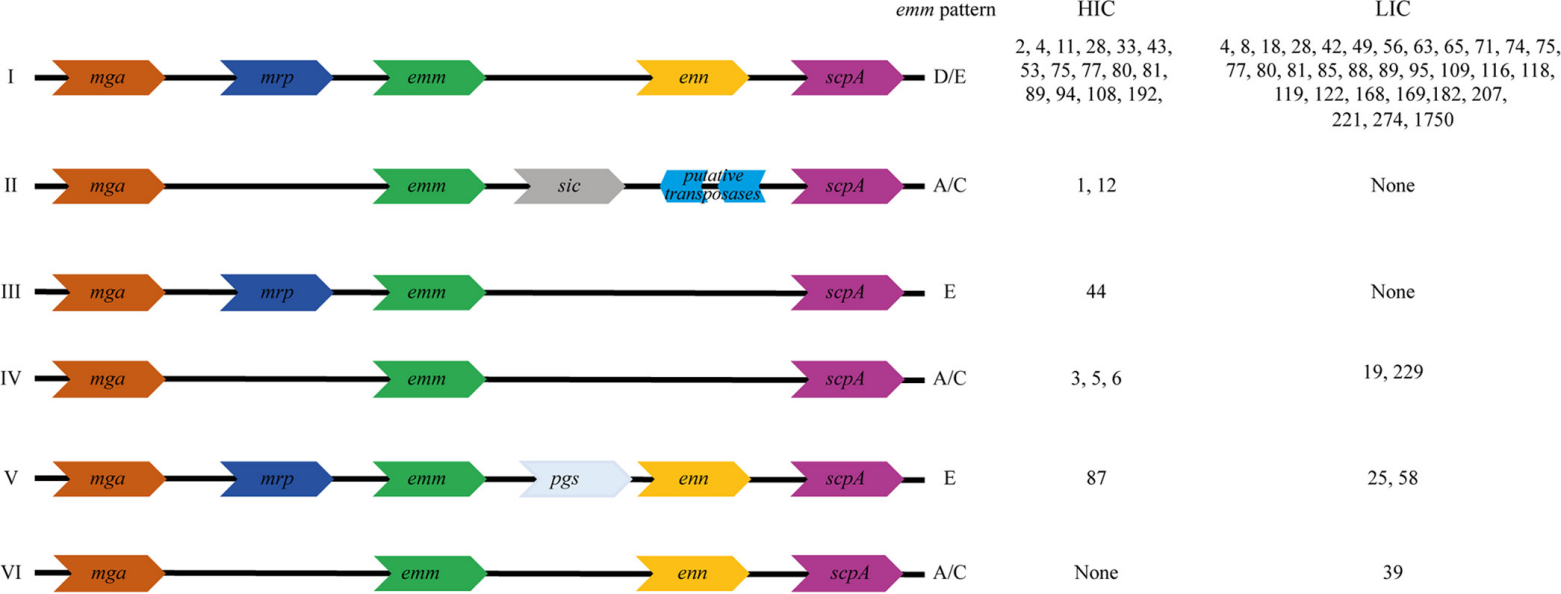
Arrangement of genes in the Mga regulon of Gambian LIC and Sheffield HIC isolates. The genes within the *mga* regulon for each isolate were determined, and Mga regulon type I to VI was assigned. The majority of *emm* types in both the Sheffield HIC isolates and the Gambian LIC isolates were type I, with the M-like protein genes *mrp* and *enn* flanking the M protein gene *emm*. The previously assigned *emm* pattern A-C, D, or E (based on the *emm* type) is also given. The streptococcal inhibitor of complement (*sic*) gene was identified only in Sheffield HIC *emm*1 isolates, and the distantly related to *sic* (*drs*) gene was found only in Sheffield HIC *emm*12 isolates. The gene *pgs* encodes Pgs, a 15.5-kDa protein of unknown function (27).

Six different Mga regulon compositions were identified across isolates from both sites (Fig. 5), but the vast majority of *emm* types from both sites were Mga regulon type I, consisting of *mga*, *mrp*, *emm*, *enn*, and *scpA.* This type was found in 31/36 *emm* types in Gambian LIC isolates and 16/23 *emm* types in Sheffield HIC isolates, accounting for 88% (75/85) and 71% (98/139) of the Gambian LIC isolates and Sheffield HIC isolates, respectively. Mga regulon type II, with the *emm*1 streptococcal inhibitor of complement (*sic*) or *emm*12 SIC-related gene (*drs*), was found only in Sheffield HIC isolates.

Alleles for *mrp* and *enn* were extracted and compared for associations with *emm* and geographical location of the isolate. Ninety-seven *mrp* genes and 92 *enn* genes were extracted from the 107 Gambian LIC isolate genomes, resulting in 44 unique *mrp* suballeles and 48 unique *enn* suballeles. From the 142 Sheffield HIC isolate genomes, we extracted 101 *mrp* genes and 99 *enn* genes, resulting in 22 unique *mrp* suballeles and 21 unique *enn* suballeles. For the majority, unique alleles were associated with *emm* type and geographical location, although phylogenetic analysis did show that overall there was limited geographical restriction between closely related alleles (Fig. S7). There were two main clades for both Mrp and Enn, each with one clade associated with E cluster *emm* patterns and the other associated with a mix of *emm* patterns. We did identify some instances of the same *mrp* allele associated with different *emm* types, although with one exception this was restricted to the Gambian LIC isolates. The *mrp*202 allele was shared by *emm*119 and *emm*162 isolates, and *mrp*60 was shared by *emm*85 and *emm*89 isolates. Suballeles *mrp*193.14 and *mrp*193.15 (same encoded amino acid sequence but different nucleotide sequences) were found in *emm*116 and *emm*86, respectively. Different suballeles of *mrp*195 were found in the Gambian LIC *emm*18, *emm*95, and *emm*/stG1750 isolates but also in Sheffield HIC *emm*53 isolates. A similar pattern was also found with *enn*, with different suballeles of *enn*199 found in the Gambian LIC *emm*65 and *emm*182 isolates and suballeles of *enn*26 found in the Gambian LIC *emm*168 but also Sheffield HIC *emm*89 isolates.

10.1128/msphere.00469-22.7 FIG S7 Phylogenetic relatedness of unique Mrp (A) and Enn (B) alleles. A maximum likelihood phylogenetic tree was generated from an amino acid alignment of unique Mrp or Enn alleles, using RAxML with 100 bootstraps (branch support shown by color scale). The Mrp or Enn allele is shown, followed by the associated *emm* type(s), *emm* cluster(s), and population, either LIC (Gambian) or HIC (Sheffield). Asterisks indicate the shared *emm* types identified in both sites. Download FIG S7, TIF file, 1.2 MB. Copyright © 2022 Bah et al. 2022 Bah et al. https://creativecommons.org/licenses/by/4.0/ This content is distributed under the terms of the Creative Commons Attribution 4.0 International license.

We also looked for the presence of the *fbaA* gene downstream of *scpA* (outside the Mga regulon), which encodes a surface protein associated with the infection potential of pattern D skin isolates (11, 28). This gene was found in all D pattern and E pattern isolates but was absent in 75% of A-C pattern isolates (Table S1).

### FCT types in the Gambian LIC and Sheffield HIC isolates.

**FIG 6:**
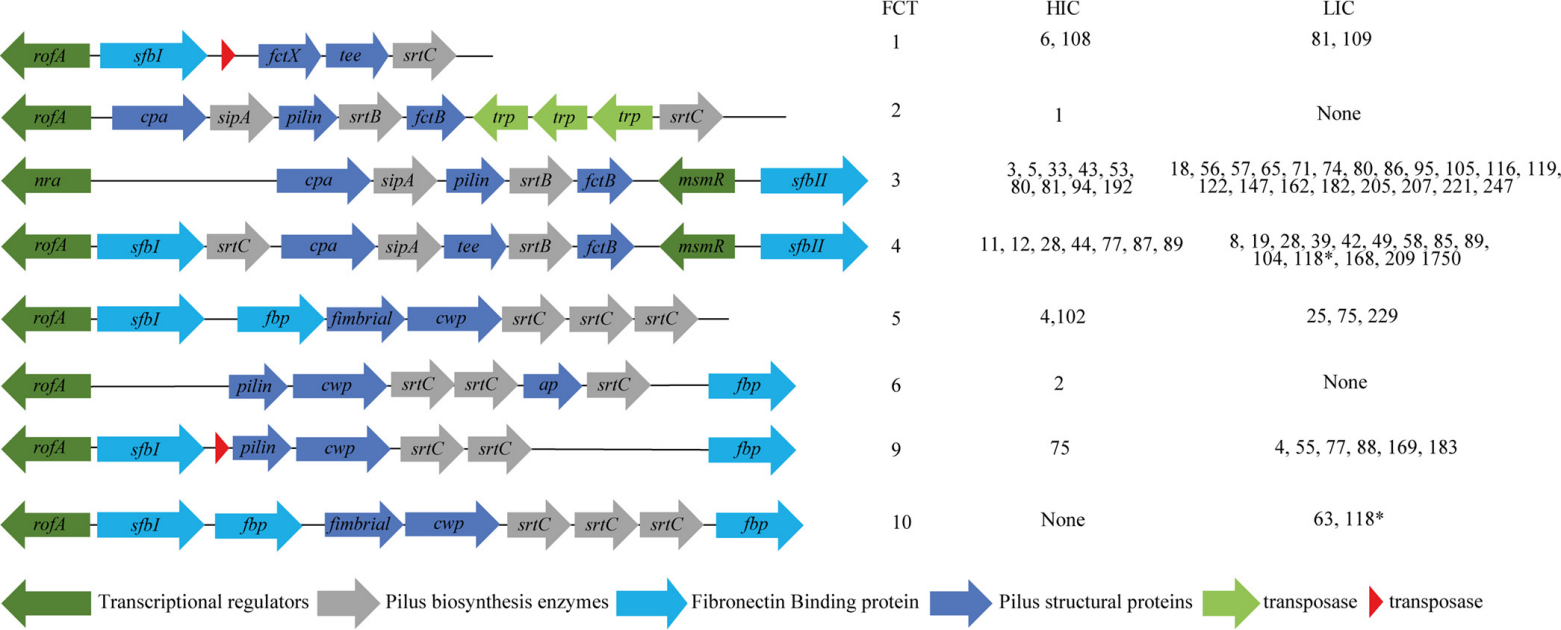
FCT arrangement patterns identified in Gambian LIC and Sheffield HIC isolates. FCT regions were extracted from *de novo* assemblies, and the FCT type was assigned based on the predicted function and order of genes within the extracted region. The *emm* types of isolates with each FCT type are shown for Sheffield HIC and Gambian LIC isolates. A new FCT region was identified (FCT10) as similar to that of FCT5 but with an additional gene encoding for a fibronectin binding protein after the sortase genes. For all *emm* types, there was at least one isolate with a designated FCT type in a single contiguous region. The only exception to this was *emm*118 (*), where the FCT was estimated to be FCT4 and the new FCT10 for each of the two isolates as the FCT region was split over two contigs. In FCT1, transposases were found in Sheffield HIC *emm*6 and *emm*108, and in FCT9, transposases were found in Sheffield HIC *emm*75 and Gambian LIC *emm*4. fbp, fibronectin binding protein; cwp, cell wall protein; ap, ancillary protein; trp, transposase.

The fibronectin-collagen binding-T antigen (FCT) region, which is classified into 9 different types (FCT1 to FCT9), comprises pilin structural and biosynthesis proteins and adhesins that could also be potential determinants for tissue tropism (29). Therefore, we investigated the diversity of the FCT regions in the skin infection isolates from The Gambia and Sheffield. Eight different patterns were identified across the two geographical sites, corresponding to FCT1 to FCT6 and FCT9, as well as a previously unidentified pattern found among the Gambian LIC isolates, which we termed FCT10; it was similar to FCT5 but with an additional fibronectin binding protein (Fig. 6). FCT3 was found in the most *emm* types in both Gambian LIC and Sheffield HIC isolate collections, 20/46 (43%) and 9/23 (39%), respectively, although this represented only 23% of the Sheffield HIC isolates compared to 41% of the Gambian LIC isolates. FCT4 was also found in a high proportion of *emm* types, accounting for 7/23 (30%) and 11/46 (24%) *emm* types, which represents 28% and 30% of Sheffield HIC and Gambian LIC isolates, respectively. Due to the prevalence of *emm*108 and *emm*1 in Sheffield HIC isolates, 33% of the isolates were either FCT1 or FCT2, whereas only 6% of the Gambian LIC isolates were FCT1 and no Gambian LIC isolates were FCT2. There was only one example of isolates of the same *emm* type with two different FCT types, and that was within the two Gambian LIC *emm*118 isolates. While one *emm*118 (ST1205) isolate was estimated to be FCT4, the other (ST354) was estimated to be FCT10, alongside the two Gambian LIC *emm*63 isolates. The FCT regions in both *emm*118 isolates, however, were estimated, as they were not found within a single contiguous sequence.

We also compared the amino acid sequences of the proteins encoded by FCT regulatory genes *rofA*, *nra*, and *msmR* and identified several variations. For the majority, variations were common to all isolates within an *emm* type and there were no obvious variations that may affect function (such as nonsense mutations). We found that 9/10 Sheffield HIC *emm*1 isolates carried three variations within RofA that characterized them as being part of the M1_UK_ lineage associated with high *speA* expression (30). No other isolates were found to carry any of these three RofA variations.

### Hyaluronic capsule biosynthesis genes.

Although the hyaluronic capsule is considered an important virulence factor that plays a role in adhesion and virulence, it was recently shown that genotypes *emm*4, *emm*22, and *emm*89 lack the *hasABC* operon required to synthesize the capsule. Additionally, in HICs, there is a high proportion of isolates within different genotypes whereby *hasA* or *hasB* has either been deleted or carries a mutation that would render the encoded protein nonfunctional, predicted to result in the lack or reduction of capsule (18). The *hasABC* operon was detected in all the Gambian LIC isolates, including the *emm*4 and *emm*89 isolates, supporting the findings that they have a different core genome compared to that of HIC *emm*4 and *emm*89 isolates, which all lacked the *hasABC* operon. No variations were detected in the *hasA* and *hasB* genes that would lead to truncated proteins in the Gambian LIC isolates, except for one *emm*74 isolate with a *hasA* variant that would encode a truncated HasA. In the Sheffield HIC isolates and consistent with previous findings (18), all *emm*28, *emm*77, and *emm*87 isolates were predicted to produce truncated HasA, and all *emm*81 and *emm*94 isolates were predicted to produce truncated HasB. Three other isolates were predicted to produce truncated HasA and a further two to produce truncated HasB, but these were sporadic examples within *emm* types (Table S1).

### Prevalence of antimicrobial resistance genes.

Of the 107 Gambian isolate genomes, the *tetM* gene encoding tetracycline resistance was identified in 79/107 (73.8%), and 37 of these (33.6% of the total population) also carried the *tetL* gene and one carried *tetK*. Furthermore, *dfrG* or *dfrK*, both encoding trimethoprim resistance, were identified in 10/107 (9.3%) and 17/107 (15.9%) isolates, respectively. Only 53/142 (37.3%) of the Sheffield HIC isolates carried the *tetM* gene, and no other resistance genes were found except for *ermA* in 8/142 (6.5%) isolates, and two *emm*11 isolates carried *ermB*, *sat4A*, and *aph3*. The prevalence of *tetM* was also low in the wider HIC isolate genomes: BSAC isolates (England/Wales), 19%; PHE isolates (United Kingdom), 8%; and ABCs isolates (United States), 14%. It was also rare in LIC Fijian isolate genomes (*n* = 6/346, 2%) but very common in LIC Kenyan isolates, at 87% (*n* = 286/328). There was also a high proportion (*n* = 153/286) of Kenyan isolates, like Gambian isolates, where *tetL* was detected alongside *tetM* (47% of the total population) (Table S2).

### Vaccine antigen diversity.

Based on the number of isolates with *emm* types present in the vaccine, the potential coverage of the 30-valent M protein vaccine for the Gambian LIC isolates was 24%, with only 11 vaccine-included *emm* types (Fig. S8). On the other hand, the potential coverage for the Sheffield HIC isolates was 61%, although only 14 were vaccine-included *emm* types. This suggests limited potential for this vaccine for low-income settings such as The Gambia, although there may be potential for cross protection, as has been seen for some *emm* types (4, 31).

10.1128/msphere.00469-22.8 FIG S8 Potential coverage of the S. pyogenes 30-valent vaccine. The percentages of Gambian LIC (A) and Sheffield HIC (B) isolates of *emm* types included in the 30-valent vaccine (black) and other *emm* types identified in each site but not included in the vaccine (red) are shown. The cumulative vaccine coverage for each site is also shown. The *emm* types without the bars are vaccine-included *emm* types but are not seen in the data set. Download FIG S8, TIF file, 1.3 MB. Copyright © 2022 Bah et al. 2022 Bah et al. https://creativecommons.org/licenses/by/4.0/ This content is distributed under the terms of the Creative Commons Attribution 4.0 International license.

Among other potential vaccine candidates, the genes *spy0651*, *spy0762*, *spy0942*, *pulA*, *oppA*, *shr*, *speB*, *adi*, *ropA*(*tf*), *spyCEP*, *slo*, *spyAD*, *fbp54*, and *scpA* were recently highlighted as conserved potential targets (16). All Gambian LIC and Sheffield HIC isolates carried all 14 genes, and BLASTp analysis indicated that all genes were highly conserved in all isolates with less than 1% sequence divergence (>99% identity) from the corresponding genes in reference genome MGAS5005 (*emm*1).

## DISCUSSION

The overall global burden of S. pyogenes infection and associated postinfection sequelae highlights the need for more research into treatment and prevention, with a particular focus on vaccine development. Maximal global impact of a preventative vaccine against S. pyogenes can be achieved only on the back of a better understanding of the global diversity of the S. pyogenes population, but to date, large-scale genomic studies have been focused mainly on HIC isolates. The Gambia, West Africa, is an LIC with a high burden of streptococcal skin infections (15). Studies on circulating *emm* types in this region, and in other African countries, indicate a much higher level of diversity than that seen in HICs (6–9), and this is reflected in the limited African genomic data (16). In this study, we aimed to contribute genomic data and provide molecular characterization of S. pyogenes in The Gambia by WGS of isolates collected during a population-based study of skin infections in children aged 5 years and under. For a comparator skin infection isolate collection, we also genome sequenced isolates from Sheffield, United Kingdom, to represent HIC isolates. We also compared some of our findings with available genomic data from other LICs and HICs.

We identified a high number of different *emm* types in the LIC isolate collection from The Gambia compared to the HIC isolate collection from Sheffield and no dominant type. This was the same for LIC isolates from Kenya and Fiji, although the majority of Gambian *emm* types were also found in either Kenya or Fiji. Previous *emm* typing data from various regions across The Gambia between 2004 and 2018 also indicated a high level of diversity. In this previous study (9), molecular typing (not WGS) methods were used and identified 69 *emm* types for 215/230 skin infection isolates (15 were nontypeable). Of these 69 *emm* types, 38 were shared with our 107 isolates. There was also a lack of a dominant *emm* type, with the most prevalent being *emm*65 (~7%), *emm*11 (~5%), and *emm*28 (~5%). Although *emm*80, *emm*85, and *emm*229 were detected in this 2004-2018 collection, albeit at lower levels (each at ~1%), no *emm*/stG1750 was identified in this collection. A further 7 *emm* types found in our 107 isolates were absent from the older collection. This suggests that in this geographical location, a high level of *emm* type diversity exists over time, but *emm* types can emerge or disappear.

In the Sheffield HIC isolates, five *emm* types (*emm*108, *emm*89, *emm*12, *emm*1, and *emm*4) accounted for ~60% of the isolates. Again, this was similar to the wider HIC isolate genome collections from the United Kingdom and the United States, although *emm*108 was rarer. There was limited overlap across Sheffield and The Gambia, with only seven shared *emm* types: *emm*4, -28, -75, -77, -80, -81, and -89. This number of shared *emm* types increased to 35 of the total 62 *emm types* when we compared the wider HIC isolate genomes with the wider LIC isolate genomes. However, for the majority, it was clear that these *emm* types represented different genetic backgrounds between geographical locations, supporting previous findings that *emm* might not be a good marker for characterizing a diverse global population (16). Our Sheffield isolates were genetically similar to other HIC isolates and clustered within lineages of other UK and U.S. isolates. Some clustering of LIC isolates from different countries was also observed. It is, however, unclear as to why there is limited sharing of isolates between HICs and LICs. Of the five most common HIC *emm* types (*emm*1, -3, -12, -28, and -89), only *emm*28 and *emm*89 were also found in LIC isolates but existed as separate distinct lineages associated with geographical origin. More work is required to fully understand the diversity within LICs and the limited genetic relatedness to HIC isolates.

A review of population-based studies (11) found that among impetigo isolates, 49.8% were D pattern, 42% were E pattern, and 8.2% were A-C pattern, compared to 1.7% D, 51.7% E, and 46.6% A-C patterns among pharyngeal isolates. This distribution is consistent with our findings in the Gambian LIC isolates (48% D, 40% E, 12% A-C) and also with the wider Gambian, Kenyan, and Fijian combined LIC isolate genome collection (40% D, 46% E, 9% A-C), although the levels of D were a bit lower in the wider population, possibly due to the range of infections included in the Kenya and Fiji collections. Both Fiji and Kenya collections included isolates from invasive and throat infections, whereas our Gambian isolates were obtained only from skin infections.

In the wider HIC isolate genome collection, we found 5% D, 45% E, and 49% A-C, which differed from our Sheffield isolate collection of 36% D, 35% E, and 29% A-C. Although we did not specifically focus on impetigo, we did select for skin infection isolates for our Sheffield isolates and seem to have captured more skin-associated D pattern isolates than throat-associated A-C pattern isolates. The wider HIC collection (BSAC, PHE, and ABCs isolates) is composed mainly of invasive isolates. Despite the higher proportion of D (skin-associated) isolates in our Sheffield collection, there was still a higher proportion of A-C (throat-associated) isolates in Sheffield than in The Gambia. The five dominant HIC *emm* types were either pharyngeal specialist pattern A-C (*emm*1 and *emm*12) or generalist pattern E (*emm*4 and *emm*89), with only *emm*108 representing skin specialist pattern D. More data are needed to support the concept of tissue tropism, particularly for HIC impetigo isolates and LIC pharyngeal isolates. Little molecular information is available for S. pyogenes causing skin infections in the United Kingdom or other HICs, as isolates are not routinely collected and typed. The dominant *emm* types found in the Sheffield HIC isolates reflected what we found in the wider HIC collection, with *emm*1, *emm*12, and *emm*89 in the top four for the United Kingdom and the United States. It is also typical of what has been found in other types of infections, with *emm*1, *emm*12, and *emm*89 leading among invasive isolates in the United Kingdom (18) and *emm*1, *emm*4, *emm*12, and *emm*89 common among UK scarlet fever cases and upper respiratory tract infections (30, 32). Very similar patterns of *emm* types causing invasive disease are also found in other European countries and North America, with *emm*1, *emm*28, *emm*89, *emm*3, *emm*12, *emm*4, and *emm*6 leading (33). The genotype *emm*108 has not previously been reported to be a common *emm* type in the United Kingdom or elsewhere but was reported in 2018/2019 by Public Health England to be a cause of national upsurges in infections in England/Wales (https://assets.publishing.service.gov.uk/government/uploads/system/uploads/attachment_data/file/800932/hpr1619_gas-sf3.pdf). The data on the prevalence of this *emm* type are based on invasive disease, as only invasive infections are notifiable in England/Wales. From the available data, it is not clear if this *emm* type would have been common among throat infections as well as skin infections during this time but suggests that it is not unique to our sampled geographical region of Sheffield, United Kingdom.

For the Gambian LIC isolates, all six E *emm* clusters were represented, with the most common being E6 (18%), closely followed by E4 (16%) and E3 (14%). E6 was recently found to be the leading cluster in Gambian noninvasive isolates (skin and pharyngeal) but with E3 leading among invasive isolates (9). D4 was also common in Gambian LIC isolates (17%) but more so in Sheffield HIC isolates, where 35% of the isolates were D4. This frequency was almost equal to that of all E clusters combined but again was explained in part by the high number of *emm*108 isolates.

As our isolates from Sheffield and The Gambia represented a skin-associated population, we compared the genomic regions previously linked to tissue tropism, namely, the Mga regulon and the FCT region. We found that the majority of Sheffield and Gambian isolates had Mga regulon pattern I, with the *emm-*like genes *mrp* and *enn*, consistent with the high number of D/E pattern isolates. Within the Sheffield HIC *emm*4 isolates, we found that 4/9 carried the *emm-enn* fusion gene, and this was also associated with degraded prophages in these isolates (34, 35). Given the high number of isolates carrying Mrp and Enn, it is possible that they contribute to pathogenesis at the same, or even greater, level as the M protein (27). The M-like proteins have not been well characterized, and their role and expression may vary depending on the allele or other genetic factors. The existence of two major clades within the Mrp and Enn phylogeny is of interest and may indicate differing domains and functions. Despite *enn* and *mrp* being adjacent to the *emm* gene, we did not observe sharing of *enn* and *mrp* alleles with *emm* type over the two geographical sites (Sheffield and The Gambia). We did, however, see the same allele or very closely related alleles of *mrp* and *enn* shared with different *emm* types across different geographical locations.

The FCT region encodes proteins essential for pilus construction, including a major pilus subunit, one or two minor subunits, and at least one specific sortase and a chaperone (36). The pili of the M1 isolate SF370 has been shown to be essential for adherence to human tonsil and human skin (37), indicating its role in primary interactions and establishment of infection. Other factors included within the FCT region are fibrinogen and fibronectin binding proteins, which may also contribute to host cell interactions, as well as transcriptional regulators. We identified the previously described FCT types FCT1 to FCT6 and FCT9 among our isolates but also a new FCT type (FCT10) that was based on FCT5 with an additional fibronectin binding protein. FCT2 and FCT6 were restricted to Sheffield HIC isolates, and the new FCT10 was found only in Gambian LIC isolates. FCT3 and FCT4 were the most common types across both sites, found in 70% (16/23) and 74% (34/46) of *emm* types, representing 54% (76/142) and 69% (74/107) of the Sheffield HIC and Gambian LIC isolates, respectively. FCT3 and FCT4 have been shown to share the greatest similarity and can undergo recombination (36). Given their prevalence in our isolates, FCT3 and FCT4 could be the most suitable for skin infection. The gene *fbaA*, which we identified as present in all isolates except for the majority of A-C pattern types, has also been found to contribute to skin infection (28). The regulator *msmR* has been shown to have a positive effect on fibronectin binding protein expression and may also control other surface proteins, affecting host cell adhesion (38). Previous work has shown that there is a high level of variability in host cell interactions and biofilm formation between isolates sharing the same FCT (39). This indicates that there are other bacterial factors involved in the expression of FCT-related genes. The role of the regulators *nra* and *rofA* does vary between isolates of differing genetic backgrounds, with evidence of environmental effects such as pH and temperature (36). We explored the sequences of *rofA*, *nra*, and *msmR* and found several different variations, but many seemed to be related to *emm* type and it is difficult to determine if any variation would affect function. This was also the case for the two-component regulator CovR/CovS and the regulator of *cov*, RocA, for which variations can affect the expression of many virulence factors. Variations in CovS and RocA were common among both Gambian LIC isolates and Sheffield HIC isolates, but the transcriptional impact of any of these amino acid changes is unclear. Only one Sheffield HIC isolate had an amino acid difference in CovR (M17I, *emm*77) and one other Sheffield HIC isolate had a premature stop codon in CovS; both may alter expression of virulence genes. Whether there are differences in expression and control of FCT and other virulence factor genes between LIC isolates and HIC isolates and/or between skin infection isolates and isolates of other types of infection is yet to be determined. The complex nature of regulatory systems can make it difficult to determine the impact of single-amino-acid variants, and control of transcription may vary between *emm* types.

In addition to analysis of tissue tropism-associated genomic regions, we also looked at genes that were unique or overrepresented in one population compared to the other. An interesting finding was the presence of a gene cluster related to the production of streptococcin A-FF22 in ~36% of Gambian LIC isolates but its absence in all Sheffield HIC isolates. It was also rare in other HIC isolates, at 1%, compared to 28% in other LIC isolates. Streptococcin A-FF22 is a linear type AII lantibiotic originally identified in strain FF22 with activity against susceptible strains of S. pyogenes as well as other streptococcal species (40). The streptococcin operon was found within a region that varied between isolates and may also include the gene *mccF*, predicted to encode a microcin C7 self-immunity protein, although its exact function is unknown. It is possible that MccF could provide protection against streptococcin A-FF22 in cases where isolates do not carry the streptococcin operon. MccF was also present in a higher proportion of Gambian LIC isolates than Sheffield HIC isolates. Whether the lactococcin region and/or MccF provides a competitive advantage over other streptococcal strains or species remains to be determined.

We also identified that 42% of LIC isolates carried a gene cluster thought to be involved in the glucuronic acid utilization pathway. This gene cluster was found in only 12% of HIC isolates. Beta-glucuronidases are enzymes capable of releasing single glucuronic acid sugars from glycosaminoglycans, including hyaluronic acid, to be used as a carbon source. They have been studied in gut bacteria, where they play an important role in human health and drug metabolism (41). It has been found that when rendering the glucuronic acid utilization nonfunctional, hyaluronic acid capsule production increased in Streptococcus equi subsp. *zooepidemicus* (24). The role for this pathway in S. pyogenes has not been studied but may allow for metabolism of sugars to which other strains or species do not have access, thereby providing a competitive advantage.

Conversely, we found a greater prevalence of the type 2A CRISPR region in HIC isolates than in LIC isolates. All isolates of the five leading Sheffield HIC *emm* types (*emm*1, -4, -12, -89, and -108) carried both CRISPR types 1C and 2A, except *emm*108 isolates, which did not carry type 1C. The type 2A system has been well studied for its role in immunity and its use as a biotechnological tool, but the impact this CRISPR and the type 1C CRISPR regions have on the wider streptococcal genome is less well studied (25). There is some evidence to suggest that CRISPR genes could be involved in genome-wide transcription regulation (42). The phage immunity that CRISPR provides may also limit the transfer of advantageous prophage-associated virulence factors, such as superantigens and DNases. However, conversely to the lack of type 2A CRISPR in LIC isolates, we found fewer prophage-associated virulence factors in LIC isolate genomes than in HIC isolate genomes, particularly DNases. Almost all (4,375/4,820, 91%) of the HIC population carried at least one prophage-associated DNase gene, whereas only 5% of LIC isolates carried *spd3* and only 19% of isolates carried *spd1*, which is associated with the superantigen *speC*. DNase genes such as *sda1* have been shown to be necessary and sufficient to degrade neutrophil extracellular traps (43), and therefore, the lack of these in LIC isolates could be suggestive of limited/reduced ability of immune evasion and warrants further investigation into their invasive capacity.

The prophage-associated *speC* and *ssa* were more common in Sheffield HIC isolates than in Gambian LIC isolates, and three Sheffield isolates carried two copies of *speC*, along with the DNase gene *spd1*, on two separate phages. Although *speA* was almost equally as common in the Gambian LIC population, all except one of the 22 *speA-*positive Gambian LIC isolates carried *speA4*, which is associated with a prophage-like element that was previously found only in *emm*6, *emm*32, *emm*67, and *emm*77 (26) rather than a complete prophage, as found in the Sheffield isolates. The presence of the *speA4* allele was also very common in the wider LIC isolate genome collections, suggesting that this finding is not limited to The Gambia.

Interestingly, we also identified a gene in one Gambian LIC isolate (*emm*65) that appeared to be a fusion of 5′ *speK* and 3′ *speM*, and since *speK* and *speM* are carried by phage, it could be a result of recombination of phages carrying the two genes. It was also identified in 13 Fijian and 7 PHE isolates. BLASTp analysis of the potential fusion protein product identified similar (2- to 3-amino-acid difference) variants in six published genomes: NS88.3 (*emm*98; GenPept accession no. PWO34032), *emm*89.14 (QCK42181), *emm*100 (QCK70992), NS426 (VGQ95836), NS76 (VGR28970), and NS6221 (VHG25078).

All of the HIC-unique genes identified by pangenome analysis were found to be prophage/transposon associated, suggesting greater prophage diversity in the Sheffield HIC isolates. It would be interesting to explore prophage genomes as well as other mobile genetic elements further, but this is notoriously difficult with short-read sequence data due to the mosaic nature of prophages and the high levels of homology which introduce breaks in the genome assembly. Long-read sequence data can support this (35) but would be required for all isolates.

We did not focus on identifying conserved genes through our pangenome analysis that could be vaccine targets. This is because a much larger and comprehensive study capturing globally diverse isolates was recently performed (16). We did confirm that the 14 candidates previously identified to be widely conserved were also conserved across our two populations. We also determined the direct coverage of the 30-valent vaccine and found it to be only 24% in the Gambian LIC population, compared to 61% in the Sheffield HIC population, although we used only *emm* type inclusion and did not explore cross-reactivity between *emm* types. The high proportion of *emm*108 in Sheffield HIC isolates was unexpected, as this was not a previously recognized dominant *emm* type, and highlights the potential for sudden and dramatic increases in new *emm* types that could escape a serotype-specific vaccine. If such a vaccine was introduced, monitoring of new variants in the noninvasive and invasive bacterial populations would be needed and on a global scale.

Our study confirms work by others (16) showing that *emm* typing alone is insufficient to comprehensively characterize global isolates. Furthermore, genetic features that have been characterized in particular HIC *emm* types, such as the absence of the *hasABC* locus in *emm*4, may not be present in LIC isolates of the same genotype. In the absence of WGS, other molecular markers, such as multilocus sequence type (MLST), *enn*, *mrp*, and FCT type, could be used in addition to *emm* typing to characterize the diverse genetic background of isolates from different geographical settings. More work is required to understand why there is such a high genetic diversity in LIC settings compared to HIC settings and with limited overlap. This may be linked to infection types, but there are insufficient data from pharyngeal infections in LICs, including The Gambia, and from skin infections in HICs. Our findings that certain gene clusters were more prevalent in isolates from one geographical location than another may indicate differential bacterial infection mechanisms. By increasing the characterization of isolates from a range of infection types over wider geographical settings, we could gain real insight into the molecular mechanisms underpinning initiation of infection, tissue tropism, and bacterial virulence.

## MATERIALS AND METHODS

### Isolates.

S. pyogenes skin pyoderma lesion isolates from 115 children under the age of 5 years in the periurban setting of Sukuta in The Gambia, collected between May and September 2018 (15), were available for WGS. As previously described, swabs were stored in liquid Amies transport medium before being taken to Medical Research Council Unit The Gambia at London School of Hygiene & Tropical Medicine (MRCG at LSHTM) for culture and identification of S. pyogenes (15). To provide a representative collection of S. pyogenes from an HIC for comparison, 160 sequentially cultured noninvasive skin and soft tissue infection (SSTI) isolates were collected from the Department of Laboratory Medicine, Northern General Hospital, Sheffield, United Kingdom, between January and April 2019. The Department of Laboratory Medicine performs microbiology diagnostics for NHS Trusts as well as primary care and community services. No patient data were obtained for these isolates, so no selection was applied for patient characteristics such as age or sex.

### Whole-genome sequencing.

Streptococcal DNA was extracted from isolates using a method previously described (44). For Gambian isolate DNA, sequencing libraries were prepared using the NEBNext Ultra II DNA library prep kit for Illumina and sequenced on an Illumina MiSeq system at MRCG. The MiSeq V3 reagent kit was used to generate 250-bp paired-end reads in accordance with the Illumina denaturation and loading recommendations, which included a 5% PhiX spike-in. Raw sequence quality assessment was performed using FastQC (v0.11.8; https://www.bioinformatics.babraham.ac.uk/projects/fastqc) with default settings, and reads were trimmed using Trimmomatic (v0.38) with the following settings: LEADING:3 TRAILING:3 SLIDINGWINDOW:4:15 MINLEN:36 (45). Sequencing of the genomic DNA from Sheffield, United Kingdom, collection isolates and a selection of isolates from the Gambian collection that were subjected to repeat sequencing after failing quality control was provided by MicrobesNG (https://microbesng.com) using the Nextera XT library prep kit (Illumina) and the Illumina HiSeq/NovaSeq platform generating 250-bp paired-end reads. Data were subjected to MicrobesNG quality control and Trimmomatic pipelines.

### WGS analysis.

*De novo* assembly was performed using SPAdes (v3.13.1) with k-mer sizes of 21, 33, 55, and 77 (46). Assembly quality statistics were generated using Quast (47) (see Table S1 in the supplemental material), and any assemblies with more than 500 contigs and a total genome size greater than 2.2 Mb were removed from downstream analyses. Coverage was estimated using an in-house script from a bedgraph file converted with bam2bedgraph from the bam file generated by mapping to the *de novo* assembly for each isolate genome using BWA-MEM (48). Coverage ranged from 9.95- to 290.45-fold (Table S1). Prokka (v1.13.3) was then used to annotate the assemblies (49), and the pangenome was determined using Panaroo 1.2.8 with the strict mode (50). Functions for unique or overrepresented genes were predicted using BLAST analysis with the NCBI database and manually checked. Single nucleotide polymorphism (SNP) distances were determined from the Panaroo core gene alignment output using snp-dists (v0.7.0; https://github.com/tseemann/snp-dists). RAxML (v8.2.12) (51) was used to generate maximum likelihood phylogenetic trees based on the core gene alignment, with a general time-reversible (GTR) substitution model and 100 bootstraps. Phylogenetic trees were visualized and annotated using iTOL (52). The *emm* types were determined from the *de novo* assemblies using emm_typer.pl (https://github.com/BenJamesMetcalf/GAS_Scripts_Reference). Where necessary, *emm* genes were manually located and the type was determined using the CDC *emm* typing database (https://cdc.gov/streplab). New *emm* subtypes were submitted to the database for assignment. Multilocus sequence types (MLSTs) were determined using the MLST database (https://pubmlst.org/spyogenes) and a script from the Sanger pathogen genomics group (https://github.com/sanger-pathogens/mlst_check). Any new alleles and sequence types were submitted to the PubMLST database.

Whole-genome sequence data for other S. pyogenes isolates were also included in this study to represent wider collections of HIC or LIC isolates (Table S2). Annotated genome assemblies for 328 isolates from Kenya (accession no. PRJEB3313) (7, 16) and 346 isolates from Fiji (accession number PRJEB2839) (16, 17) were obtained from NCBI as representative LIC isolates. Genome data for 344 isolates collected by the British Society for Antimicrobial Chemotherapy (BSAC) across England/Wales (18), 2,898 isolates collected from across the United Kingdom by Public Health England (PHE) (19), and 1,436 isolates collected across the United States by the Active Bacterial Core surveillance (ABCs) (20) were used as representative HIC isolates. These data were previously assembled, annotated, and curated (18). The *emm* type and ST for each isolate were previously determined (7, 16, 18–20). Further analyses, including pangenome and phylogeny, were performed as described above. Simpson’s diversity index was calculated as previously described (9).

### Variable factor typing.

The presence of superantigen genes *speA*, *speC*, *speG*, *speH*, *speI*, *speJ*, *speK*, *speL*, *speM*, *speQ*, *speR*, *ssa*, and *smeZ* and DNase genes *sda1*, *sda2*, *sdn*, *spd1*, *spd3*, and *spd4* were initially determined by a BLAST (100% coverage, 70% identity) search of representative gene sequences against the Gambian and Sheffield *de novo* assemblies. Gene presence was then additionally confirmed by BWA-MEM (48) mapping of the short-read sequences to a pseudosequence of concatenated superantigen and DNase genes; coverage of at least 10 reads across the whole gene was used to confirm presence. Where the BLAST and mapping results did not agree, the results were manually inspected in the annotated *de novo* assemblies. For detection of the superantigen and DNase genes in the wider collection of LIC and HIC isolates, a BLAST search only was performed.

Antimicrobial resistance (AMR) gene carriage was determined with ABRicate v0.8.13 (https://github.com/tseemann/abricate) using the ARG-ANNOT database (53), with a minimum coverage of 70% and a percent identity of 75%.

The nucleotide sequences for *covR*, *covS*, and *rocA* regulatory genes and the *hasA*, *hasB*, and *hasC* capsule biosynthesis genes from the S. pyogenes H293 reference genome (*emm*89; GenBank accession no. NZ_HG316453.1) were used as queries in blastn searches against the Gambian and Sheffield *de novo* assemblies. The start and end coordinates of the best BLAST hits were converted into BED files and used to extract the nucleotide sequences from the *de novo* assemblies using BEDTools (v2.27.1) (54). Extracted gene sequences were then translated into amino acids, and variants were determined in comparison to the corresponding amino acid sequences of the reference (H293) proteins. For *hasABC*, only nonsense variants and gene absence were recorded (Table S1).

### *emm* pattern and FCT regions.

To determine the *emm* pattern in the genome of each Gambian and Sheffield isolate, *in silico* PCR (https://github.com/simonrharris/in_silico_pcr) was used to extract the sequence of the whole *mga* regulon (the beginning of *mga* to the end of *scpA*) from *de novo* assemblies and then the sequence was annotated with Prokka. To improve assemblies where the *mga* regulon was not within a contiguous sequence, *de novo* assemblies were ordered against a complete reference genome of the same *emm* type (where available) using ABACAS (55) and the *in silico* PCR was repeated. An *emm* pattern of I, II, III, IV, V, or VI was assigned using BLAST to identify genes, followed by visual determination of gene location within the regulon. For 22 Gambian LIC and 3 Sheffield HIC isolates, the *emm* pattern could not be determined since a contiguous sequence for the *mga* regulon could not be obtained (detailed in Table S1).

Alleles of the *emm-*like genes *enn* and *mrp* were assigned by comparison to those identified by Frost et al. (27), ensuring 100% nucleotide identity across the entire gene sequence. Where we could not obtain a contiguous sequence for the *mga* regulon, *enn* and *mrp* alleles were determined by a BLAST search of each allele sequence against the entire *de novo* assembly. New alleles for *enn* and *mrp* were kindly assigned by Pierre Smeesters and Anne Botteaux. In some cases, breaks in the *de novo* assemblies occurred within the *enn* gene and therefore alleles could not be confirmed (detailed in Table S1).

To determine the arrangement of the genes in the FCT region and the FCT type, *in silico* PCR was used to extract the FCT region, which was then annotated with Prokka. Assemblies in which amplicons were not obtained due to contig break in the FCT regions were again ordered against a close reference of the same *emm* type (where available). The open reading frames (ORFs) within each extracted FCT region were subjected to a BLAST search against the entire NCBI database, and in combination with the order of the genes, the FCT types were assigned based on previously assigned FCT type where possible (56). For some isolates, it was not possible to obtain a contiguous sequence for the FCT region and so the FCT type was estimated based on manual inspection of the *de novo* assembly and identification of FCT-associated genes by using BLAST.

### Data availability.

Short-read sequence data were submitted to the Sequence Read Archive under accession numbers provided in Table S1 in the supplemental material.
