# Supplementary figures and images for: Genomic Characterization of Skin and Soft Tissue Streptococcus pyogenes Isolates from a Low-Income and a High-Income Setting
Source: mSphere. 2022 Dec 12;8(1):e00469-22. doi: 10.1128/msphere.00469-22 (PMC9942559; doi:10.1128/msphere.00469-22)

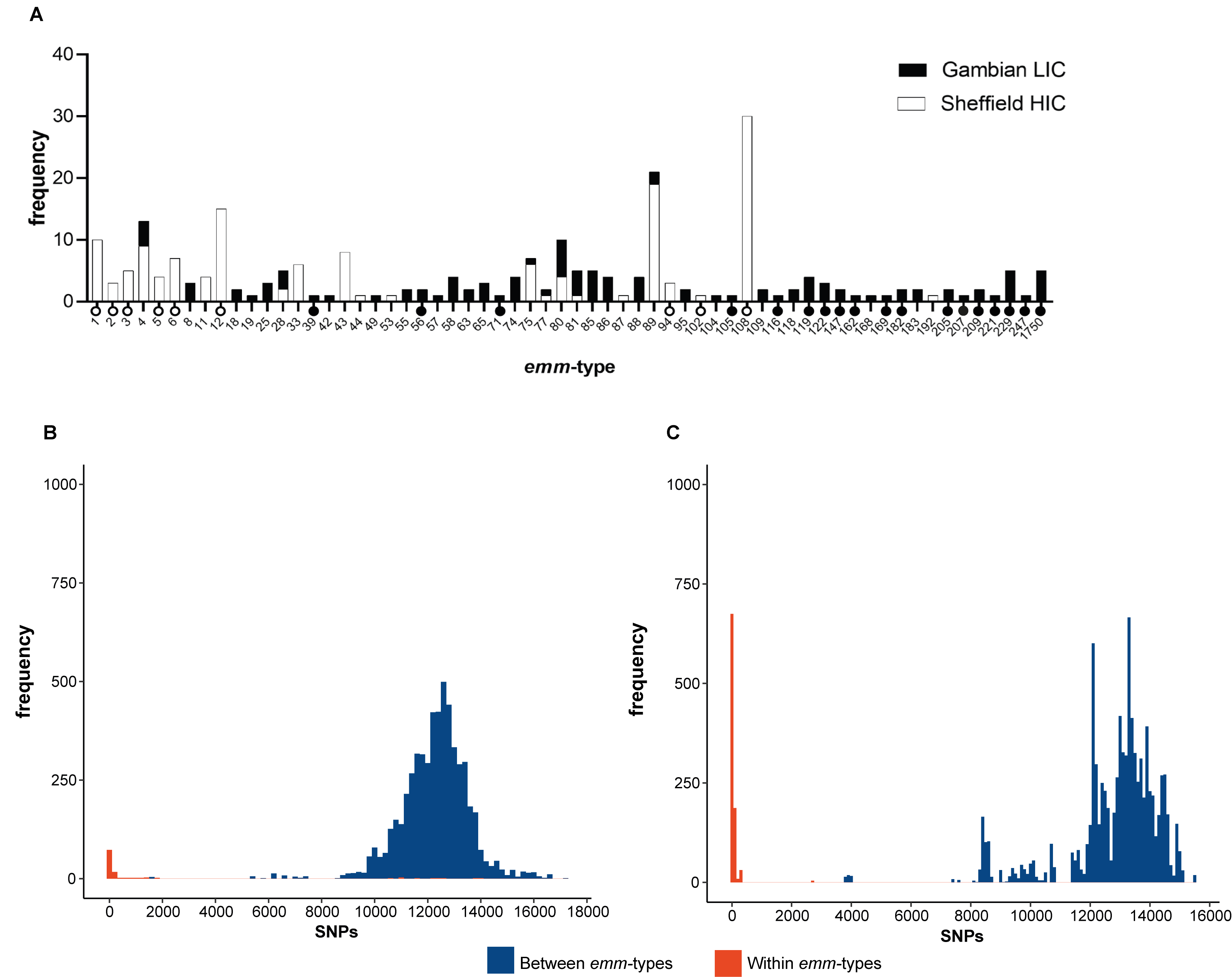

Supplement: FIG S1 [file msphere.00469-22-s0003.tif]

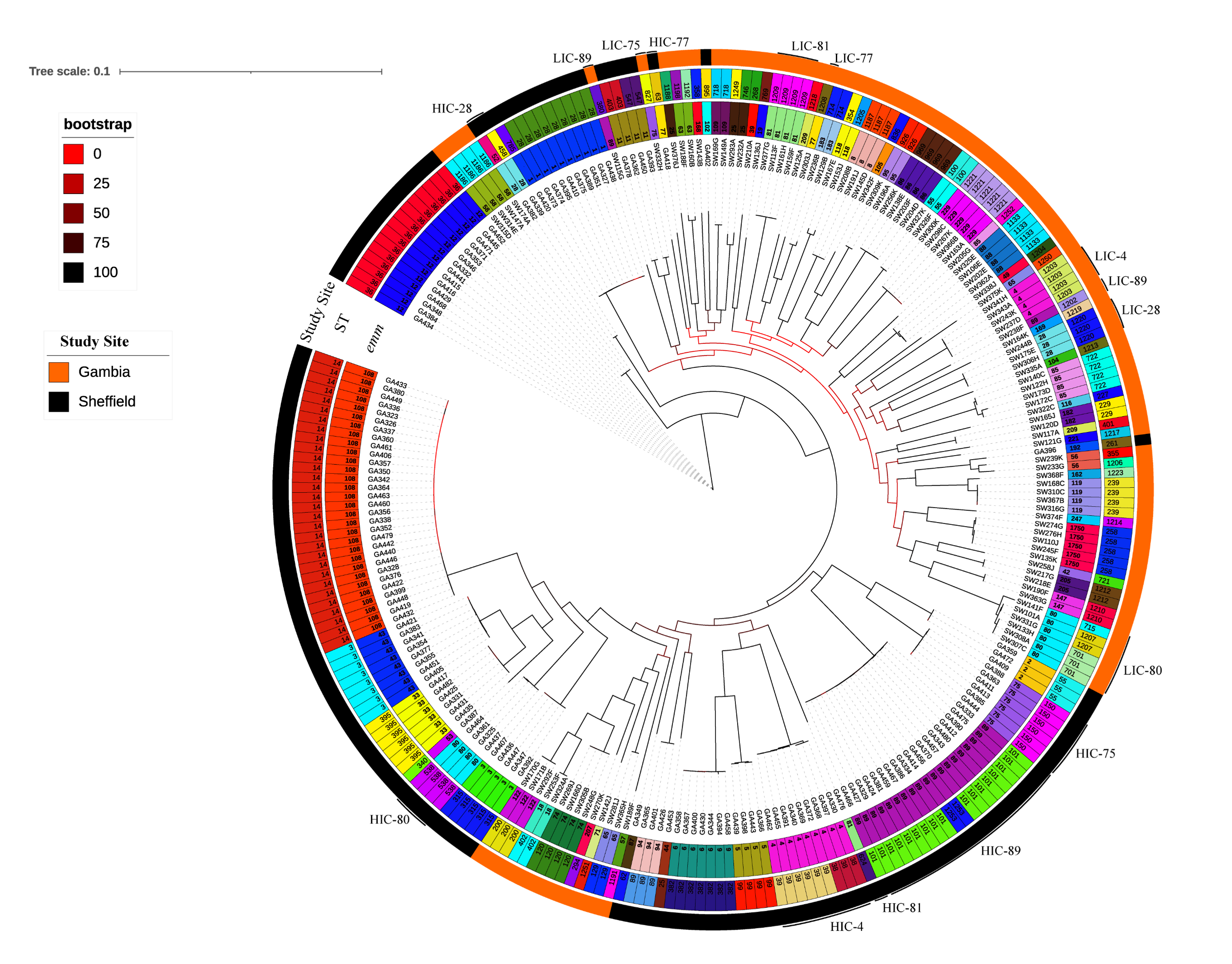

Supplement: FIG S2 [file msphere.00469-22-s0004.tif]

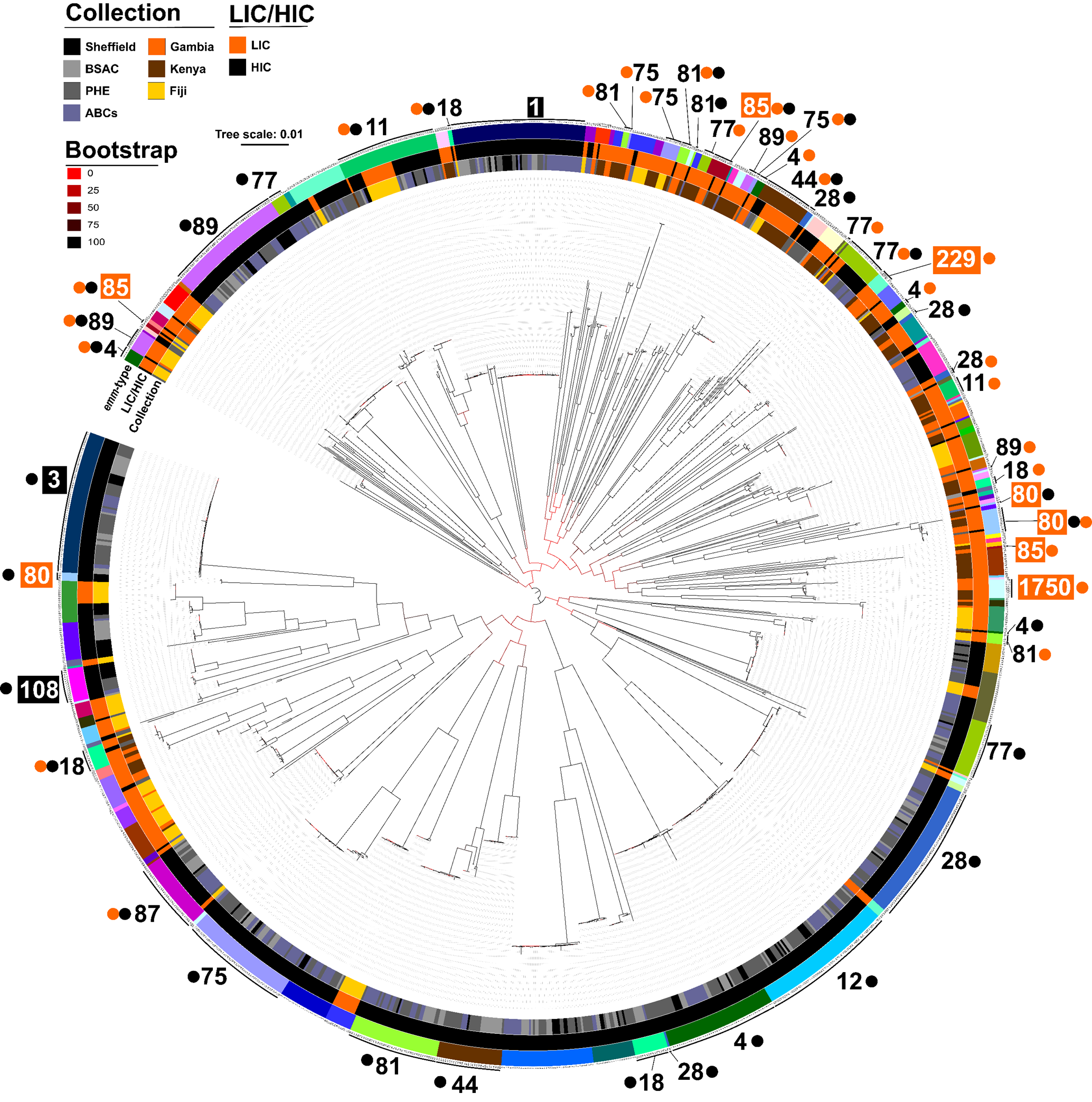

Supplement: FIG S3 [file msphere.00469-22-s0005.tif]

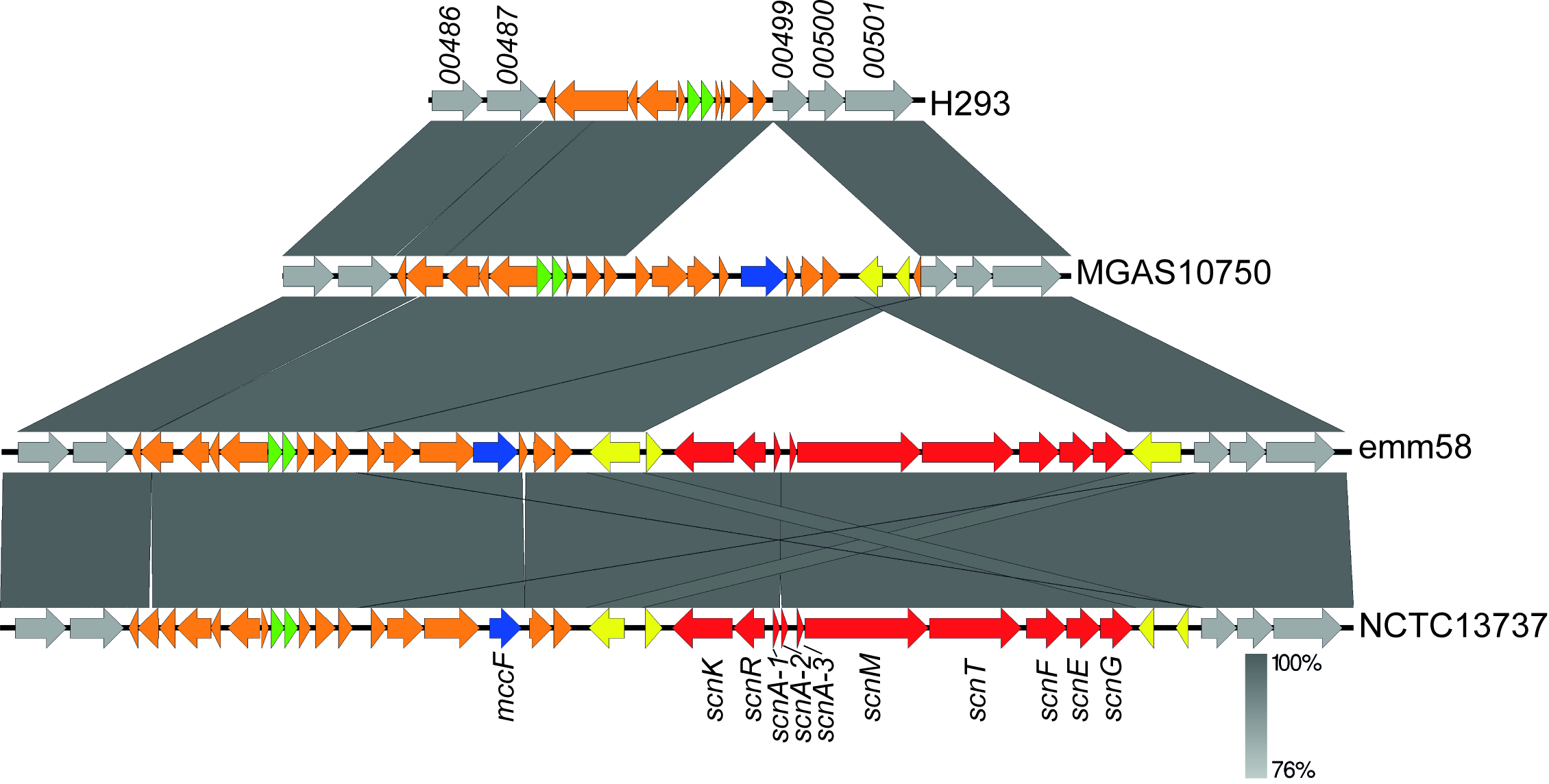

Supplement: FIG S4 [file msphere.00469-22-s0006.tif]

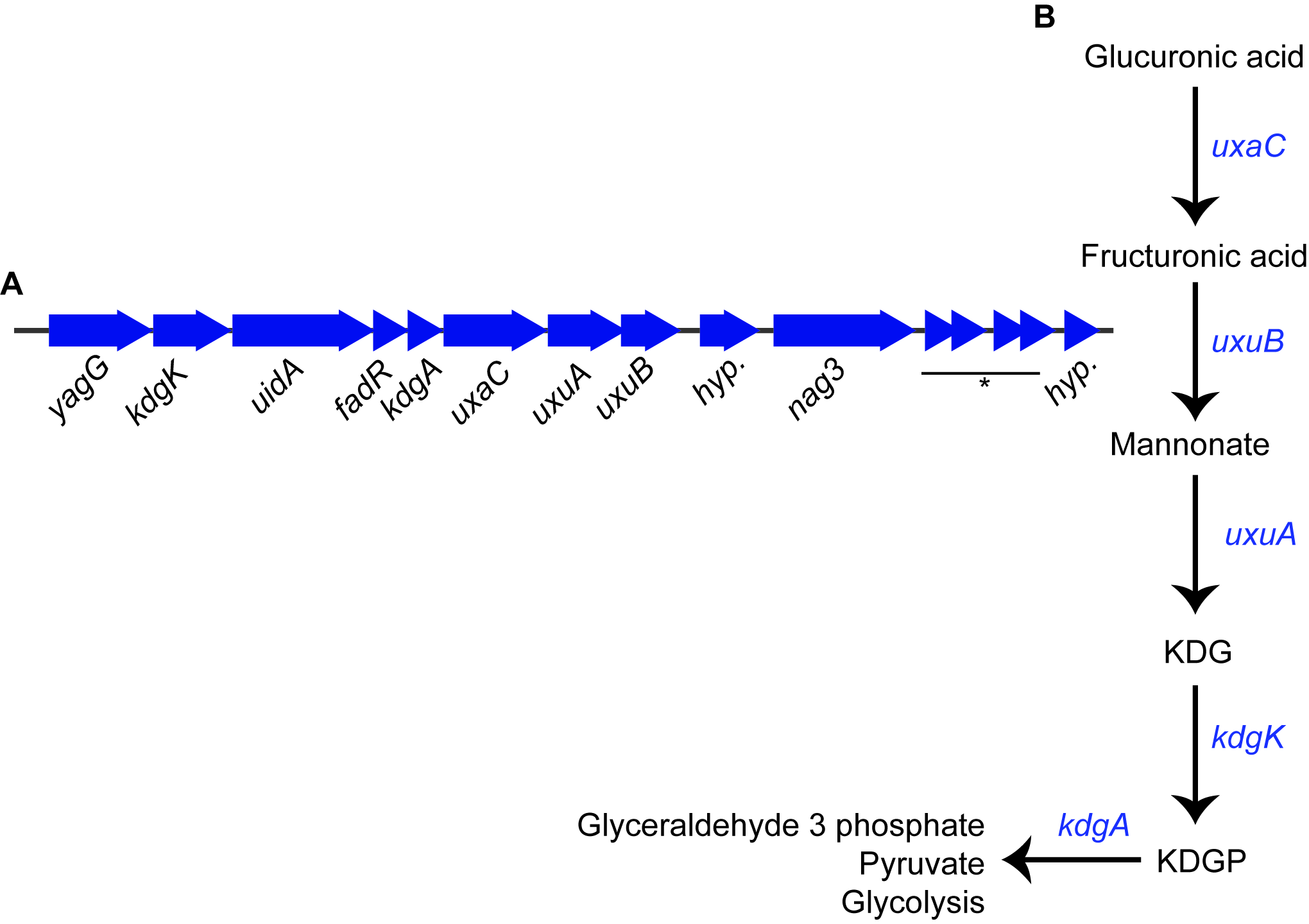

Supplement: FIG S5 [file msphere.00469-22-s0007.tif]

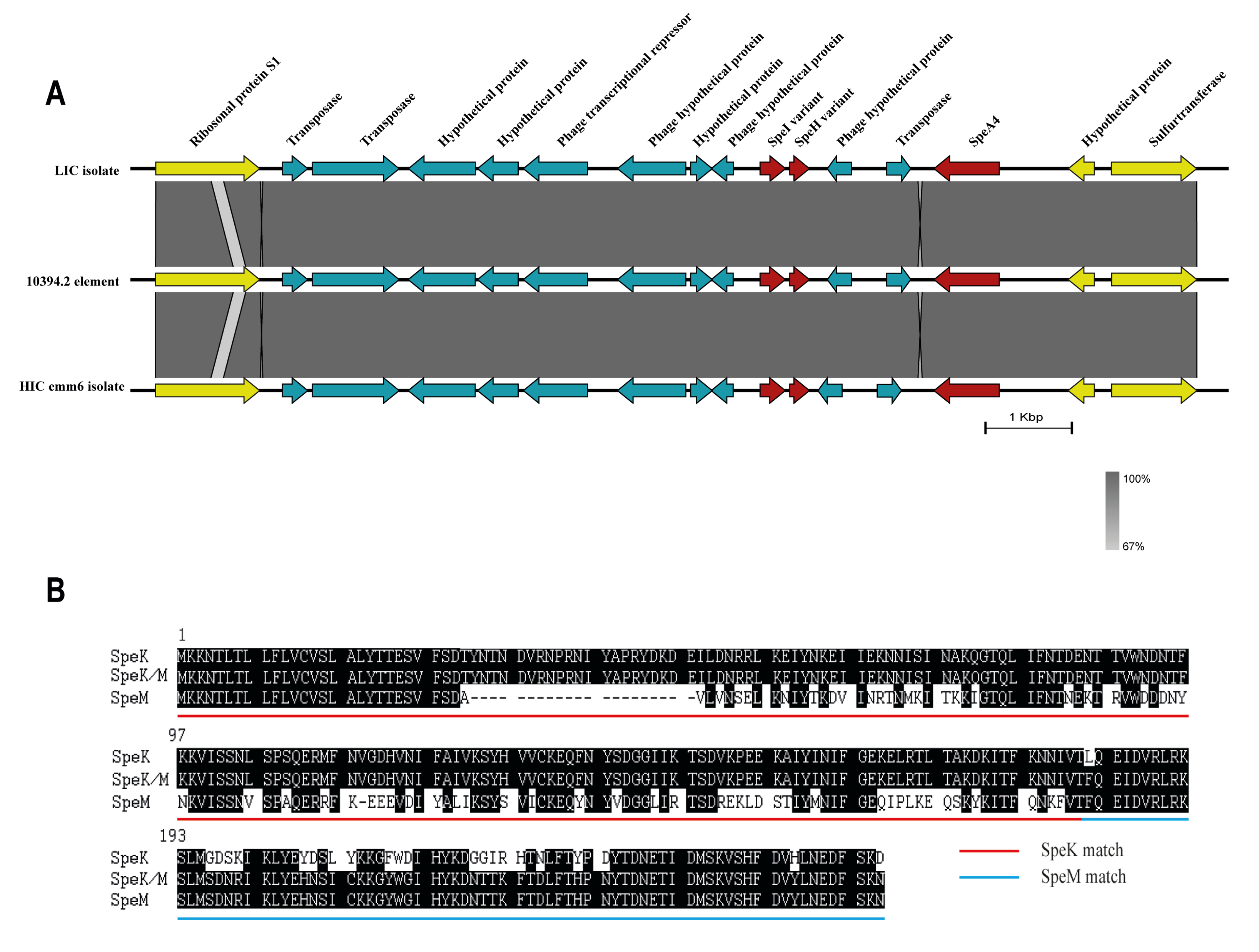

Supplement: FIG S6 [file msphere.00469-22-s0008.tif]

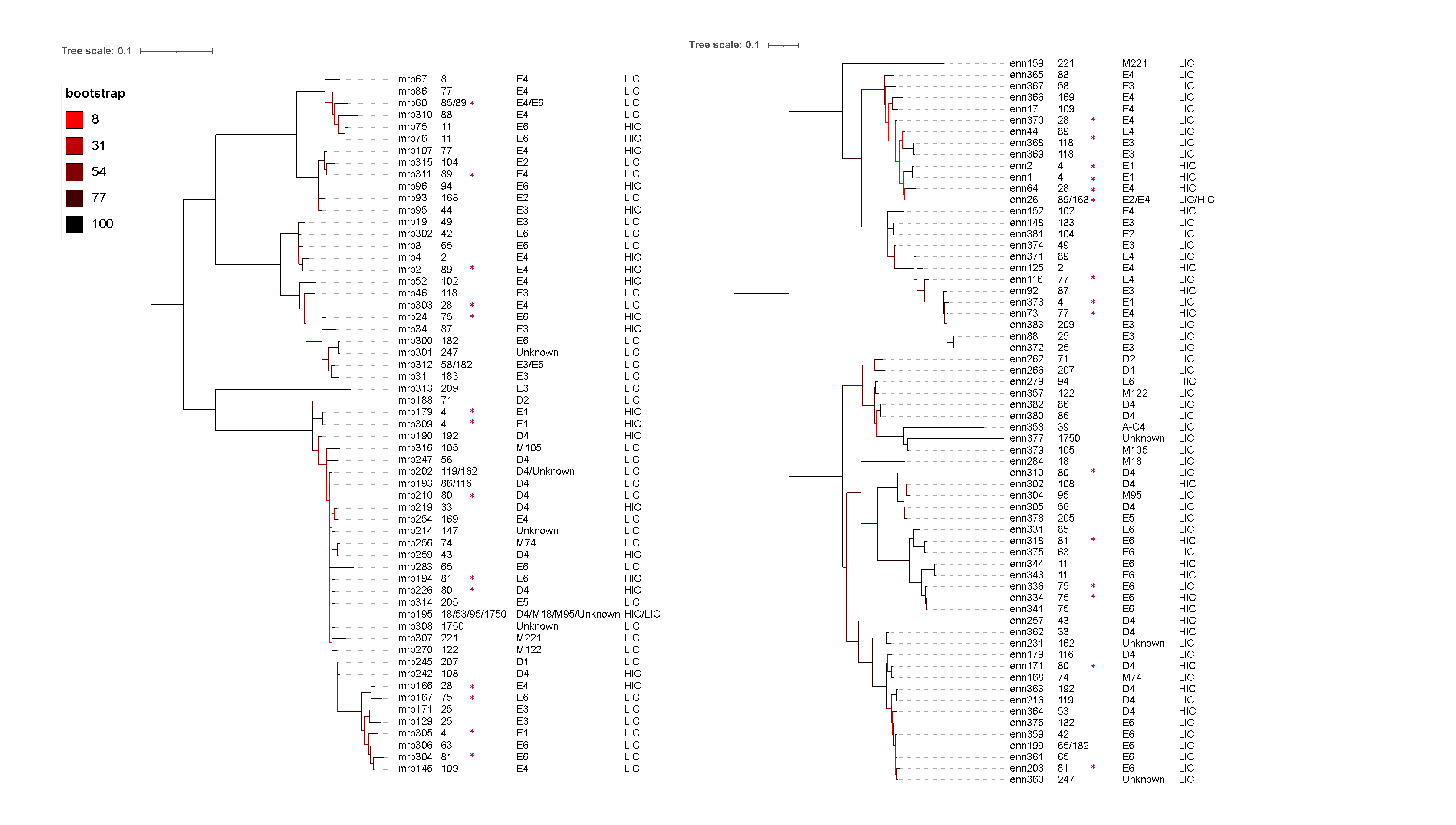

Supplement: FIG S7 [file msphere.00469-22-s0009.tif]

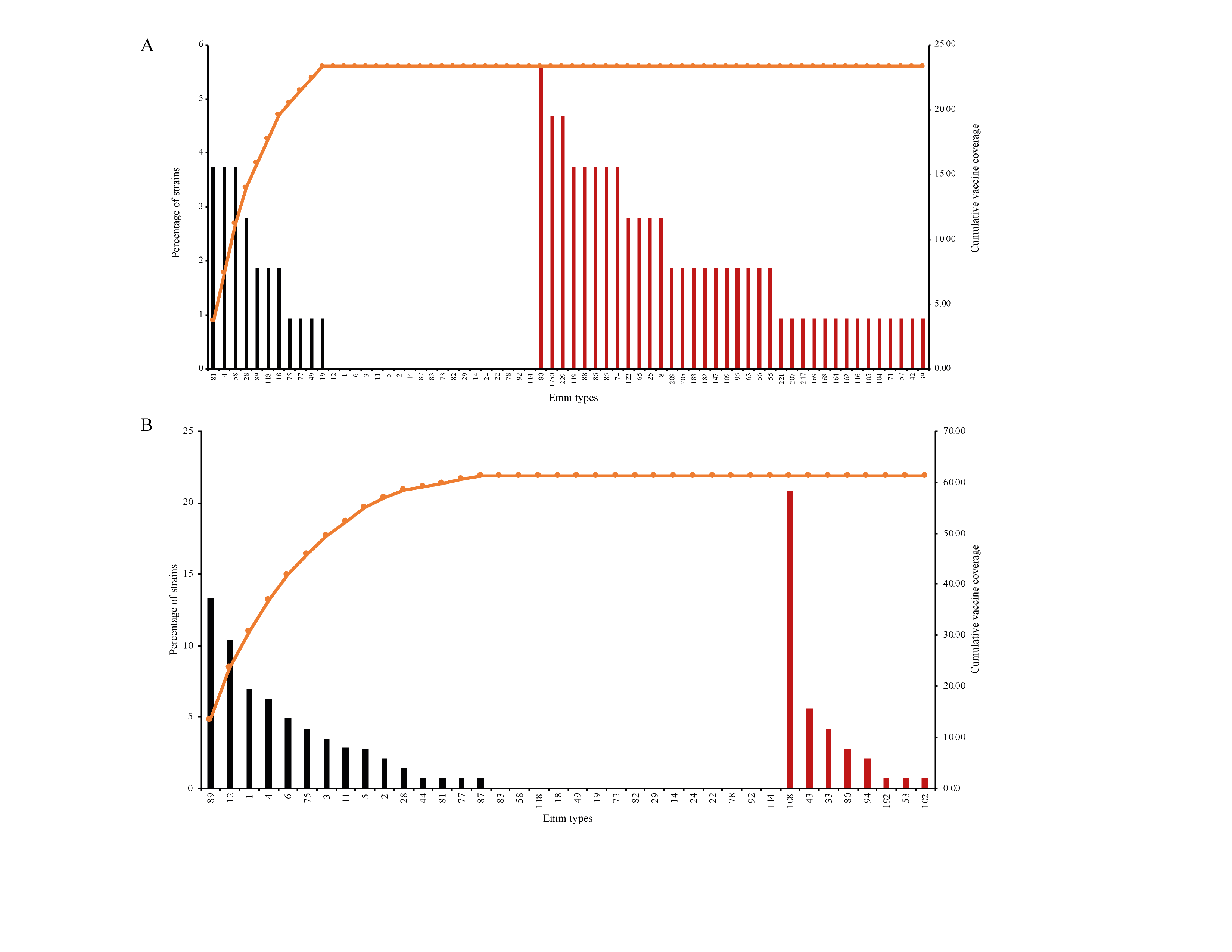

Supplement: FIG S8 [file msphere.00469-22-s0010.tif]
